# Topically applied pH-responsive nanogels for alkyl radical-based therapy against psoriasiform hyperplasia

**DOI:** 10.1080/10717544.2023.2245169

**Published:** 2023-08-10

**Authors:** G.R. Nirmal, Chia-Chih Liao, Zih-Chan Lin, Abdullah Alshetaili, Erica Hwang, Shih-Chun Yang, Jia-You Fang

**Affiliations:** a Graduate Institute of Biomedical Sciences, Chang Gung University, Kweishan, Taiwan; b Department of Anesthesiology, Chang Gung Memorial Hospital at Linkou, Taoyuan, Taiwan; c School of Medicine, College of Medicine, Chang Gung University, Taoyuan, Taiwan; d Chronic Diseases and Health Promotion Research Center, Chang Gung University of Science and Technology, Puzi, Taiwan; e Department of Pharmaceutics, College of Pharmacy, Prince Sattam Bin Abdulaziz University, Al Kharj, Saudi Arabia; f Department of Dermatology, Yale School of Medicine, Yale University, New Haven, CT, USA; g Department of Microbiology, Soochow University, Taipei, Taiwan; h Pharmaceutics Laboratory, Graduate Institute of Natural Products, Chang Gung University, Kweishan, Taiwan; i Research Center for Food and Cosmetic Safety and Research Center for Chinese Herbal Medicine, Chang Gung University of Science and Technology, Kweishan, Taiwan

**Keywords:** Nanogel, chitosan, pH-responsiveness, skin delivery, alkyl radical, psoriasis

## Abstract

Phototherapy is a conventional antipsoriatic approach based on oxygen-relevant generation of oxidative stress to inhibit keratinocyte hyperproliferation. However, this therapy can be restricted due to local hypoxia in psoriatic lesions. The generation of alkyl radicals is oxygen-independent and suppresses hyperproliferation. Herein, we established alkyl radical-based therapy to treat psoriatic hyperplasia. Because alkyl radicals are short-lived compounds, we loaded 2,2'-azobis[2-(2-imidazolin-2-yl)propane] dihydrochloride (AIPH) as a precursor of alkyl radicals into the chitosan nanogels to improve stability. The present study presented a topically applied nanogel that led to a pH-responsive network sensitive to skin pH. This pH responsiveness of the nanogels allowed fast alkyl radical release in the target site. The physicochemical properties of the prepared nanogels were determined through size, zeta potential, scanning electron microscopy, and absorption spectroscopy. The antipsoriatic activity was examined with keratinocyte- and animal-based studies. The nanogels displayed a smooth and spherical morphology with a hydrodynamic diameter of 215 nm. This size was largely increased as the environmental pH increased to 6. The nanogels heated at 44 °C produced alkyl radicals to induce keratinocyte death through the necrosis pathway. Bioimaging demonstrated that topically applied nanogels could deliver alkyl radicals into the epidermis. This targeting was accompanied by the accumulation of free radicals in the epidermis according to the 2′,7′-dichlorodihydrofluorescein diacetate assay. The imiquimod-stimulated psoriasiform animal model indicated a remarkable reduction in erythema, scaling, and overexpressed cytokines upon topical treatment of the nanogels. The transepidermal water loss of the psoriasiform skin was inhibited from 51.7 to 27.0 g/m^2^/h, suggesting barrier function recovery by the nanocarriers. The nanogels lowered hyperplasia by decreasing the epidermal thickness from 212 to 89 μm. The incorporation of 8-hydroxypyrene-1,3,6-trisulfonic acid (HPTS) as a pH-sensitive fluorescence dye in the nanogels could be used to diagnose the severity of the psoriasiform plaque due to the stronger fluorescence of HPTS in skin with lower pH (psoriasiform skin pH = 4.4) than in healthy skin (pH = 4.9). It was possible to deliver the prepared nanogels into the epidermis to restrain hyperplasia without causing cutaneous irritation.

## GRAPHICAL ABSTRACT

**Figure:**
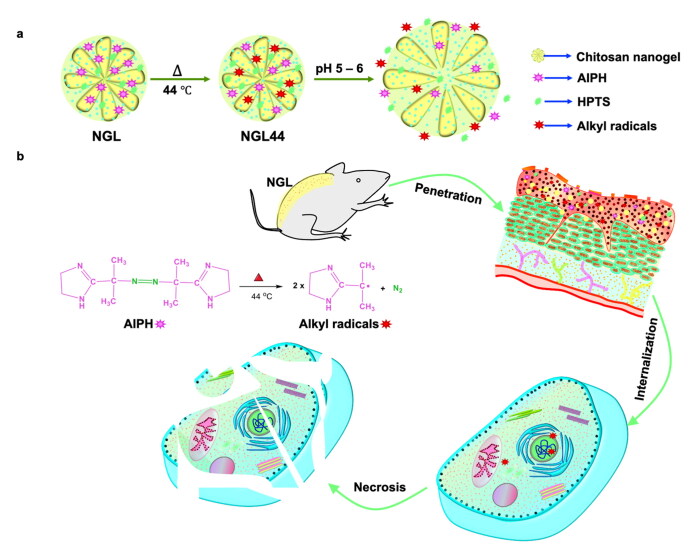

## 1. Introduction

Psoriasis is a chronic and recurrent skin inflammatory disease characterized by epidermal hyperproliferation and immune cell infiltration. It is clinically manifested as red plaque with a silver scale. The proliferation dysregulation and maturation of epidermal keratinocytes in psoriasis can result in acanthosis, parakeratosis, and hypergranulosis (Rapalli et al., 2020). Most psoriasis is categorized as mild-to-moderate severity (Knuckles et al., 2018). Topical therapy is still the main treatment for these patients. Topical phototherapies, such as psoralen and ultraviolet A (PUVA) and photodynamic therapy (PDT), can suppress psoriasis by decreasing keratinocyte proliferation. Although phototherapy is effective in treating psoriasis, the treatment results in some adverse effects, including pigmentation, photoaging, cataracts, and carcinogenesis (Almutawa et al., 2013). The need for an extra device for light irradiation is another inconvenience. An alternative effective and safer treatment for epidermal hyperplasia regression is warranted. Nanoparticle-based drug delivery opens a new field of antipsoriatic therapy with enhanced efficacy and reduced toxicity compared with conventional treatment (Petit et al., 2021). Nanoparticles serve as drug carriers that enable targeted delivery, improved safety, and theranostic application by incorporating diagnostic agents. Notably, most nanocarriers penetrate only superficial skin because of their larger size compared with free drug molecules (Pukale et al., 2020). Nanoparticle delivery to the viable epidermis and dermis is not easy. Only nanoparticles with a size of <20 nm can permeate the intact stratum corneum (SC), although the disintegrated barrier function of diseased skin leads to the transport of larger nanoparticles (Rapalli et al., 2020). Thus, a special design of nanoformulations is important for successful topical therapy against psoriasis.

Some diseases necessitate specific drug delivery regions and timing to manifest maximum effectiveness. Nanogels are a promising approach to achieve this purpose (Pinelli et al., 2020). Nanogels are defined as the aqueous dispersion of water-swollen nanoparticles formed by chemically or physically crosslinked networks of hydrophilic polymers. They are largely applied for advanced drug delivery systems due to their facile drug encapsulation, large surface area, stable dispersity, high biocompatibility, and rapid water exchange (Rajput et al., 2020). With the features of hydrogels, the nanogels maintain hydrated conditions and a shrinking–swelling nature under different environments. Nanogels are more responsive to various stimuli, such as pH, temperature, and redox, than other drug delivery systems due to their unique 3D structure (Yin et al., 2020). The skin consists of different layers with diverse pH environments. The bulk pH levels of the SC and viable skin are estimated to be 4.0–4.5 and 5.0–7.0, respectively (Plasencia et al., 2007). As the target site of keratinocyte proliferation inhibition for antipsoriatic therapy is the epidermis, we intended to design a nanogel with pH-dependent shrinking–swelling behavior that can retain smaller size for facile penetration and then swell in the epidermis to produce more hydrophilic particles for increased partitioning into viable skin (epidermis and dermis). Since viable skin exhibits more hydrophilic features than lipophilic SC with abundant lipid bilayers, the increase in nanoparticle hydrophilicity is favorable for partitioning to epidermis to target keratinocytes. The swelling also induces drug release from the nanogels to cause keratinocyte death. The pH-responsive nanogels selected in this study for accomplishing this aim were composed of chitosan. Chitosan is a kind of natural cationic polyaminosaccharide extensively developed as a drug and gene carrier due to its fascinating characteristics of biocompatibility, pH sensitivity, and possible functionalization (Wang et al., 2017b). The SC displays a negative charge on its surface, and cationic chitosan is beneficial for offering penetration into the superficial skin due to electrostatic attraction.

PUVA and PDT rely on oxygen-dependent generation of oxidative stress to activate keratinocyte apoptosis in psoriasis (Correia et al., 2021). However, abnormal keratinocyte proliferation in psoriasis contributes to local hypoxia (Tao et al., 2008; Li et al., 2017). A previous study (Pham et al., 2021) also verified concurrent inflammation and hypoxia due to immune cell recruitment in tissues. Phototherapy may result in unsatisfactory outcomes under hypoxic conditions. Another concern is the existence of natural antioxidants such as glutathione in keratinocytes (Campione et al., 2022). It is expected that the antioxidants residing in keratinocytes may compromise the effect of oxygen-dependent phototherapy. The generation of alkyl free radicals can be oxygen-independent. Cytotoxic alkyl radicals are useful for hypoxic tumor treatment through the induction of cancer cell apoptosis or necrosis (Lee et al., 2021). As alkyl radicals are highly reactive and short-lived compounds (Wang et al., 2021), they are not suitable for direct incorporation into formulations. 2,2′-Azobis[2-(2-imidazolin-2-yl)propane] dihydrochloride (AIPH) is a thermal initiator used as a radical source to release alkyl radicals after heating. AIPH is decomposed to yield two alkyl radicals upon thermal stimulation (Yang et al., 2022). AIPH demonstrates low stability in storage, and alkyl radical production at body temperature is limited (Wu et al., 2021). Nanoencapsulation can protect AIPH from decomposition (Thirunavukkarasu et al., 2019). We aimed to load AIPH as a free radical generator into chitosan nanogels to inhibit keratinocyte proliferation in psoriasis. The nanogels were heated at 44 °C to produce alkyl radicals before topical administration. The pH responsiveness of chitosan nanogels allows permeation across the SC and into the epidermis, leading to alkyl radical release in the target site.

In addition to therapeutic goals, nanoparticles can act as image-guided diagnosis platforms. In the present study, we also loaded 8-hydroxypyrene-1,3,6-trisulfonic acid (HPTS) as a fluorescent dye for the noninvasive diagnosis of psoriasis. HPTS is a pH-sensitive dye with high photostability and low toxicity (Han and Burgess, 2010). The major drawback of HPTS application is its low permeability across the cell membrane (Cao et al., 2021). Nanoencapsulation is capable of improving the intracellular internalization of HPTS. We anticipated that negatively charged HPTS would interact with chitosan, showing high entrapment efficiency. Psoriatic skin in patients has an overall lower pH (5.2) than healthy skin (5.6) (Cannavo et al., 2017). The variation in the fluorescence of pH-responsive HPTS with respect to different skin conditions is expected. To this end, we fabricated chitosan nanogels loaded with AIPH and HPTS by an ionic gelation method. This pH-responsive nanosystem was applied as a topical theranostic for controlled delivery to mitigate psoriasis hyperplasia. After physicochemical characterization, the antipsoriatic effect of the nanogels was examined in cell- and animal-based studies.

## 2. Materials and methods

### 2.1. Preparation of nanogels

Chitosan (100 mg, medium molecular weight, 75–85% deacetylation, Sigma–Aldrich) and acetic acid (6 mL) were mixed and stirred at 700 rpm for 10 min, followed by the addition of double-distilled water (6 mL) with a stirring rate of 1000 rpm. The stirring process was continued for 10 min until the chitosan was fully dissolved. Double-distilled water (2 mL) containing AIPH (5 mg), HPTS (2 mg), and sodium tripolyphosphate (10 mg) was added to the chitosan solution under stirring at 1000 rpm. Gelation occurred within 5 min after this addition. The obtained mixture was centrifuged at 11,000 rpm and 4 °C for 15 min. After removing the supernatant, the precipitate was washed three times with double-distilled water. After centrifugation, the nanogels were agitated by a probe-type sonicator at an amplitude of 80% and pulse on/off interval of 5 s:1 s at 4 °C for 1 min. The probe sonication was repeated two times with a cooling interval of 5 min between each sonication. To produce alkyl radicals from AIPH, the nanogels were heated at 44 °C (NGL44) in a dry bath incubator for 1 h before treatment of cells or animals (Supplementary Figure 1).

### 2.2. Physicochemical characterization of the nanogels

The hydrodynamic diameter and zeta potential of the chitosan nanogels were estimated by dynamic light scattering (Malvern Nano ZS90). Before the measurement, the nanogels were diluted with double-distilled water (1:100) adjusted to different pH values. To observe the size and morphology of the nanogels, the nanosystems were positioned on a carbon-film-coated copper grid and stained with phosphotungstic acid for scanning electron microscopy (SEM, Hitachi S5000). The absorbance spectra of the chitosan nanogels were studied using UV spectroscopy (Hitachi U1900). The fluorescence spectra of the nanogels were detected using a fluorescence spectrometer (Hitachi F2500). The excitation wavelength was set at 455 nm for scanning emission wavelengths ranging from 450 to 700 nm. The encapsulation percentage of AIPH and HPTS was calculated by using the ultracentrifugation method to separate the entrapped compounds from the free forms. The nanoparticles were centrifuged at 48,000× *g* and 4 °C for 40 min. The free compounds in the supernatant and entrapped compounds in the precipitate were analyzed by UV spectrophotometry at 356 nm (for AIPH) and 404 nm (for HPTS), respectively (Lin et al., 2019).

### 2.3. Free radical formation in nanogels

The 2,2′-azino-bis(3-ethylbenzothiazoline-6-sulfonic acid) (ABTS) assay was utilized to confirm the formation of alkyl radicals after heating the nanogels at 44 °C. Potassium persulfate (140 mM, 88 μL) was added to the ABTS solution (7 mM, 5 mL). After a 16-h incubation in the dark, this stock solution was diluted with water (1:44). The nanogels diluted with water (1:4, 500 μL) were mixed with ABTS solution (500 μL), followed by UV spectroscopy to record the absorbance of ABTS^+^. The free radical release from the nanogels diluted in pH 4.5 or pH 6.5 solution was evaluated by dialysis membrane with a molecular weight cutoff of 3.5 kDa (Biomate). The dialysis bag was immersed in pH 6 buffer (10 mL) and shaken for 24 h. The receptor medium was collected and analyzed by an ABTS assay.

### 2.4. Biocompatibility of the nanogels

Keratinocytes (HaCaT, AddexBio) were cultured in DMEM at a cell density of 1 × 10^5^ cells/mL and incubated at 37 °C for 24 h. Nanogels at different concentrations were added to the cell suspension, followed by incubation for 24 h. 3-(4,5-Dimethylthiazol-2-yl)-2,5-diphenyltetrazolium bromide (MTT, 0.5 mg/mL) was added to the cell suspension and incubated for 4 h. Keratinocyte viability was assessed by spectroscopic analysis at 570 nm.

### 2.5. Nanogel uptake by keratinocytes

AIPH (62.5 μg/mL) and HPTS (25 μg/mL) were incorporated into the nanogels as fluorescence dyes to observe internalization into HaCaT cells. The cells (1 × 10^5^ cells/mL) were treated with nanogels for 24 h. After removing the culture medium and washing three times with PBS, flow cytometry (ThermoFisher Attune NxT) was utilized to determine the level of nanogel internalization in keratinocytes. To examine pH-dependent HPTS fluorescence, the collected cells were dispersed in aqueous solutions with different pH values (5, 6, and 7.4) for flow cytometric analysis.

### 2.6. Intracellular alkyl radical detection

Confocal microscopy and flow cytometry were used to determine the presence of alkyl radicals in keratinocytes after nanogel treatment. The cells (1 × 10^5^ cells/mL) were treated with the nanogels (with 62.5 μg/mL AIPH and 25 μg/mL HPTS) at 37 °C for 24 h. Subsequently, the HaCaT cells were incubated with 2′,7′-dichlorodihydrofluorescein diacetate (DCFDA, 25 mM) for 45 min in the dark. The cells were incubated with 4′,6-diamidino-2-phenylindole (DAPI) for 1 h, followed by fixation with formaldehyde for confocal imaging. The flow cytometric assay was conducted by adding DCFDA into trypsinized keratinocytes and incubating for 45 min.

### 2.7. Nanogel-induced keratinocyte death

Keratinocyte death induced by the nanogels with and without heating at 44 °C was evaluated by both MTT assay and flow cytometry. After incubation with the nanogels at 37 °C for 24 h, HaCaT cells were collected for the MTT assay. Keratinocyte death induced by the nanogels was also estimated by annexin V and propidium iodide (PI) staining and flow cytometry. After nanogel treatment for 24 h, the cells were washed, trypsinized, and placed in a flow tube. Annexin V and PI (5 μL) were added to the tube for a 20-min incubation in the dark before flow cytometric analysis. Western blotting was performed to determine high mobility group box 1 (HMGB1) to verify the ­mechanism of cell death. Immunoblotting was performed according to a previous study (Nirmal et al., 2021). Glyceraldehyde-3-phosphate dehydrogenase (GAPDH) was employed as the loading control antibody to normalize the protein level.

### 2.8. Animals

One-week-old pigs were obtained from PigModel Animal Technology (Miaoli, Taiwan). Eight-week-old Balb/c hairy mice were purchased from the National Laboratory Animal Center (Taipei, Taiwan). All animal experiments were performed strictly in accordance with the Guidelines for the Care and Use of Laboratory Animals of Chang Gung University. The protocol was approved by the Institutional Animal Care and Use Committee.

### 2.9. In vitro skin absorption of nanogels

The skin from the dorsal area of the pigs was excised. The number of pigs used in the study was 6. The pig skin was mounted between the donor and receptor of the Franz diffusion cell with the SC facing the donor. The receptor was loaded with pH 7.4 buffer (5.5 mL). Then, 300 μL of nanogels (containing 62.5 μg/mL AIPH and 25 μg/mL HPTS) or free HPTS (25 μg/mL) was added to the donor compartment. The permeation region and stirring rate in the receptor were 0.785 cm^2^ and 600 rpm, respectively. After 24 h, the skin was removed from the Franz cell to examine the amount of HPTS in the skin. The skin sample was weighed, diced, and homogenized in water (1 mL) by a MagNA Lyser (Roche). After homogenization, the supernatant was collected after centrifugation at 10,000 ×*g* for 10 min. The HPTS absorption in the skin was detected by UV spectroscopy at 404 nm.

### 2.10. In vivo psoriasiform dermatitis study

The total number of mice used in the study was 24 (6 animals for each treatment groups). The Balb/c mouse back was topically treated with imiquimod (IMQ) cream (Aldara, Inova) at 62.5 mg for 5 days to evoke psoriasis-like lesions (van der Fits et al., 2009). Before IMQ activation, nanogels (100 μL) containing 62.5 μg/mL AIPH and 25 μg/mL HPTS with or without heating were applied to the dorsal skin. The phenotypic skin surface was monitored by a digital camera and hand-held microscopy (M&T Optics). The combined score (erythema, scaling, and skin thickness) was estimated to reveal the inflammation severity (scale 0–12) according to the Psoriasis Area and Severity Index (PASI) scoring. The erythema (a*) and transdermal water loss (TEWL) of the skin were estimated by colorimetry (Yokogawa CD100) and a Tewameter (Courage & Khazaka TM300). An *in vivo* imaging system (IVIS) was used to visualize the cutaneous fluorescence derived from the topically applied nanogels. The nanocarriers were administered to the skin each day for 5 days. The IVIS (PerkinElmer IVIS Lumina LT) was used to image the dorsal skin 24 h postadministration. The excitation and emission wavelengths were set at 430 nm and 445–490 nm, respectively, for IVIS to image the HPTS fluorescence.

### 2.11. Enzyme-linked immunosorbent assay (ELISA)

The cytokines in the mouse skin with or without nanogel treatment were quantified by ELISA. The punch biopsies from the skin were incubated with PBS in the presence of protease inhibitor, followed by homogenization at 6500 rpm for 30 s. The samples were centrifuged at 13,000 rpm at 4 °C for 10 min. The levels of tumor necrosis factor-α (TNF-α), interleukin-1β (IL-8), interferon-γ (IFN-γ), and IL-6 were determined by ELISA MAX Deluxe Sets (BioLegend) according to the manufacturer’s protocol.

### 2.12. Histological observation

The mouse skin was deposited into 10% buffered formaldehyde, embedded in paraffin wax, and sliced at 5 μm for hematoxylin and eosin (H&E) staining. The unstained samples were incubated with anti-Ki67, anti-Ly6G, anti-8-hydroxy-2-deoxyguanosine (8-OHDG), anti-HMGB1, or anti-cyclophilin antibodies for 1 h, followed by washing with 0.5% Tween 20 in saline. The specimens were then incubated with biotinylated donkey anti-goat immunoglobulin G for 20 min. All sections for immunohistochemistry (IHC) were visualized by optical or fluorescence microscopy. We used AlphaView software (ProteinSimple) to quantify the antibody-labeled sections.

### 2.13. In vivo skin distribution of nanogels

The total number of mice used in the study was 24 (6 animals for each treatment groups). The *in vivo* skin permeation of the nanogels was verified by confocal imaging. The excised skin from the mouse was immersed in buffered formaldehyde and embedded in paraffin wax. After sectioning, the skin slice was stained with DAPI. The skin was monitored by confocal microscopy to observe the nanogel distribution based on HPTS fluorescence. The presence of free radicals in the skin after nanogel application was visualized by DCFDA assay. The skin sections were incubated with DCFDA for 4 h in the dark. After staining with DAPI for 1 h, the samples were imaged via confocal microscopy.

### 2.14. Safety of nanogels on mouse skin

The total number of mice used in the study was 18 (6 animals for each treatment groups). The safety of the use of the chitosan nanoparticles on mouse skin was inspected in healthy mice. The nanogels (200 μL) containing 62.5 μg/mL AIPH and 25 μg/mL HPTS were topically administered to the dorsal skin of the mouse. This procedure was repeated for 5 days. After removing the nanogels, the skin was examined for erythema and TEWL. After a 5-day treatment, skin surface imaging and H&E-stained histology were evaluated.

### 2.15. Statistical assay

The significant differences in the data of different treatment groups were compared using the Kruskal–Wallis test, while the post hoc test conducted to check the individual differences was Dunn’s test. The statistically significant levels of probability included *p* < .05 (*), 0.01 (**), and .001 (***).

## 3. Results

### 3.1. Physicochemical characterization of the nanogels

**Figure 1.**
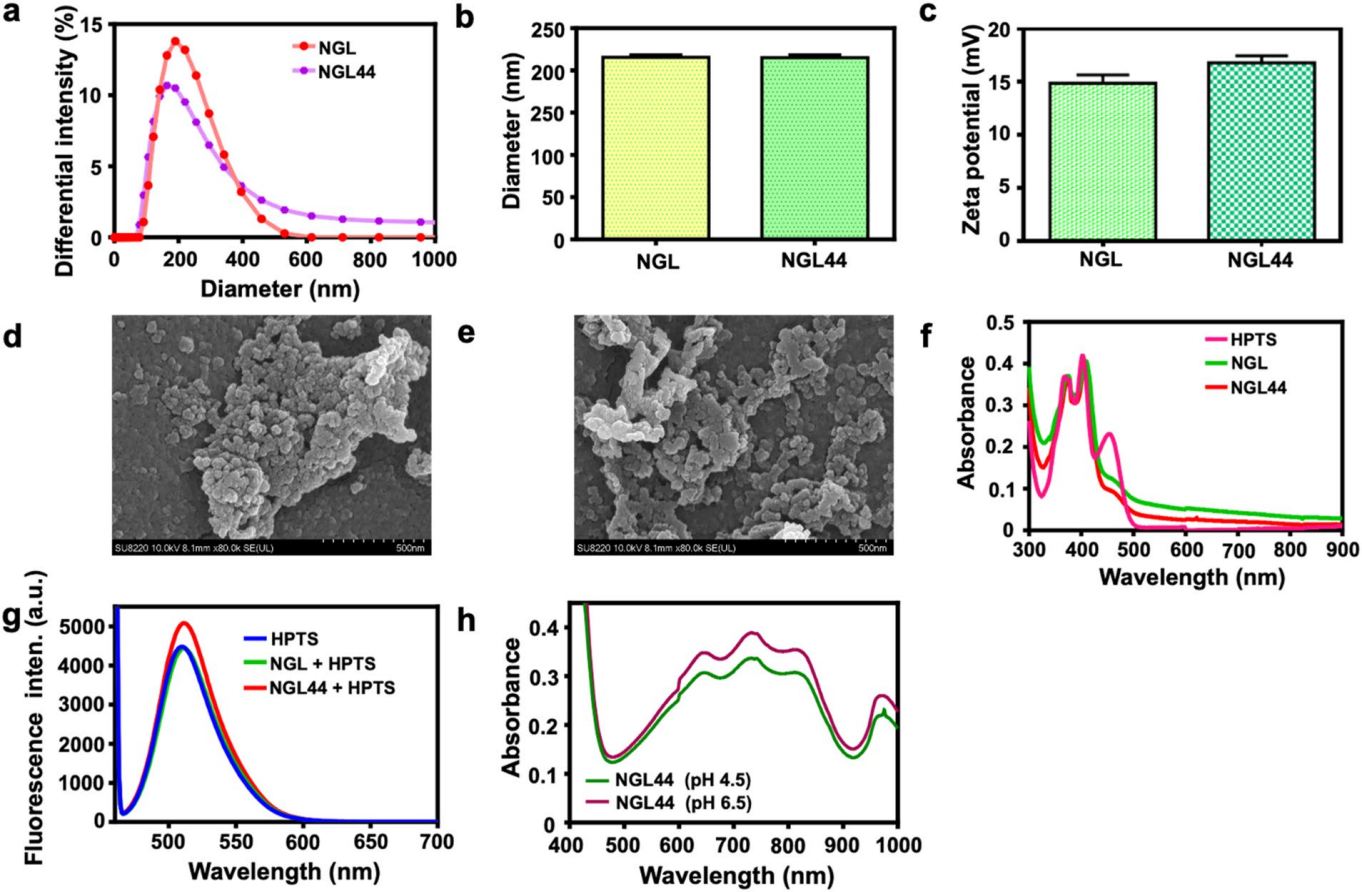
Physicochemical characteristic analysis of the chitosan nanogels: (a) dynamic light scattering analysis on size distribution of NGL and NGL44; (b) Hydrodynamic diameter evaluation of NGL and NGL44; (c) zeta potential of NGL and NGL44; (d) SEM visualization of NGL; (e) SEM visualization of NGL44; (f) absorption spectra of the NGL, NGL44, and free HPTS; (g) fluorescence emission spectra of NGL and NGL44 studied using fluorescence spectroscopy at excitation wavelength of 455 nm; (g) pH-dependent alkyl radical release (determined by ABTS assay) from NGL44. The experiment was performed for 24 h at pH 4.5 and pH 6 loading nanogel 44 into dialysis membrane bag. Data are expressed as mean and   SD (*n* = 3).

The hydrodynamic size of the nanogels with or without 44 °C heating was examined first. The sizes of both nanosystems showed a unimodal distribution (Figure 1(a)). The average hydrodynamic diameter of NGL was 215.6 nm (Figure 1(b)). After heating at 44 °C (NGL44), the size displayed no obvious change (215.1 nm). A positive surface charge of both nanoformulations was detected due to the cationic charge of chitosan (Figure 1(c)). There was no significant difference between the zeta potentials of NGL and NGL44, although a higher surface charge was obtained after heating. To observe the shape of the nanogels, NGL and NGL44 were characterized by SEM (Figure 1(d,e)). The nanoparticles aggregated while coating the SEM grid. However, we could still visualize monodispersed nanogels with a smooth and distinct spherical form. The particle diameters of NGL and NGL44 in SEM were calculated to be 42.5 and 41.0 nm, respectively (Supplementary Figure 2(A–C)). The diameter of the nanogels calculated from the SEM micrograph was much smaller than that measured by the hydrodynamic form. The detection of hydrodynamic size was conducted in an aqueous environment, a situation in which the results are usually larger than the genuine diameter. As the nanogels were observed in the dry state for SEM imaging, significant shrinkage of the nanogels occurred. To investigate the effect of the environmental pH change on nanogel responsiveness, the hydrodynamic size and zeta potential were measured at different pH values (Supplementary Table 1). Both nanoformulations (NGL and NGL44) showed an average size and zeta potential of approximately 215 nm and 28 mV in double-distilled water (pH 5.5), respectively. The polydispersity index of the nanogels at pH 5.5 (in double-distilled water) was approximately 0.3. The size increased to approximately 8500 nm as the pH increased to 6 and 7.4, while the zeta potential decreased to 3–5 mV. The SEM image also exhibited a significant enlargement of nanogel size after the increase of pH to 6 (Suppl. Fig. 2D). The Zetasizer instrument can only provide accurate measurement of particle diameters between 0.3 and 5000 nm. Although this instrument can still estimate particle sizes of 5000–10,000 nm, this measurement is vague. However, the inaccurate size determined at pH 6 and 7.4 still indicated that the nanogels largely aggregated when transferring from acidic to neutral/basic environments. This indicated swelling of the nanogels at a pH of 6. A pH reduction to 5 resulted in hydrodynamic sizes of 286 and 269 nm for NGL and NGL44, respectively. The zeta potential detected in pH 5 solution was comparable to that in double-distilled water (pH 5.5). The encapsulation percentages of AIPH (62.5 μg/mL) and HPTS (25 μg/mL) were 91.8 ± 2.0% and 87.6 ± 2.7%, respectively. This indicated the high loading efficiency of both compounds by the nanogels.

**Figure 2.**
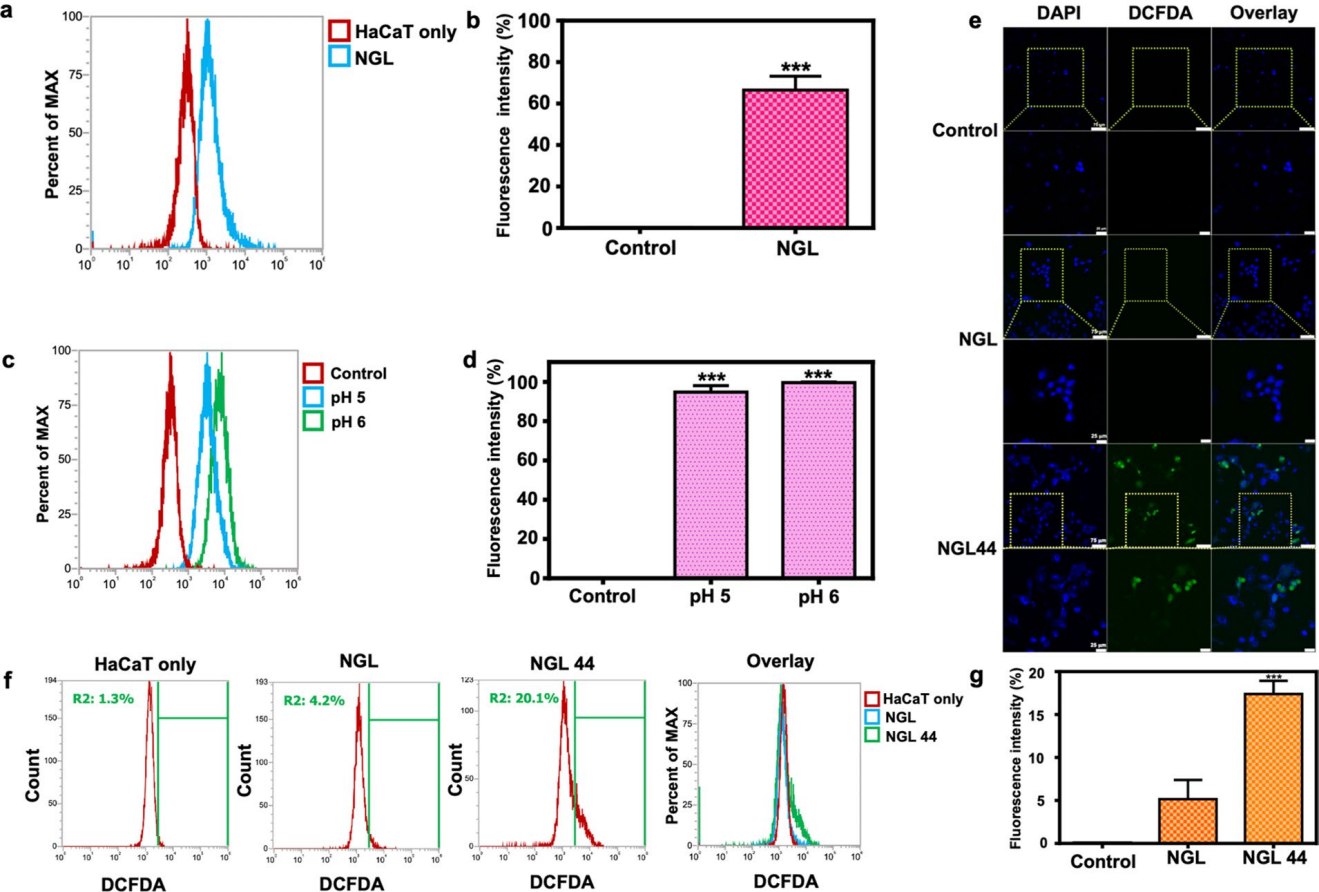
Nanogel internalization and alkyl radical presence in keratinocytes: (a, b) flow cytometric analysis representing nanogel internalization into human keratinocytes; (c, d) evaluation of nanogel fluorescence in keratinocytes at weak acidic conditions of pH 5 and pH 6 through flow cytometry; (e) confocal microscopic visualization for the presence of alkyl radicals in keratinocytes. DCFDA was employed as an alkyl radical indicator; (f, g) Flow cytometric analysis for alkyl radical in keratinocytes treated with NGL and NGL44. All data are expressed as mean and   SEM (*n* = 3). ***p < .001, compared with respective control groups. DCFDA: 2′,7′-dichlorodihydrofluorescein diacetate.

The optical properties of the HPTS-loaded nanogels were determined by UV–visible absorption and fluorescence spectroscopy. The UV–visible spectrum of HPTS in double-distilled water displayed absorption peaks at 377, 404, and 459 nm (Figure 1(f)). The absorption spectra of NGL and NGL44 revealed the characteristic peaks of HPTS, indicating successful entrapment into the nanogels. HPTS reveals two distinct absorption signals near 405 and 455 nm belonging to protonated and unprotonated forms, respectively. The HPTS absorbance at 459 nm was decreased after nanoencapsulation, which could be due to the deprotonation of HPTS in the nanogels. The fluorescence spectrum showed that free HPTS was strongly fluorescent at 508 nm with excitation at 455 nm. The spectrum of the nanogels showed an emission peak near 508 nm, corresponding to the signal of HPTS. The generation of alkyl radicals in the nanogels after heating was tested by employing ABTS as the probe. Upon reacting with a free radical, ABTS produces ABTS^+•^ with strong absorption at 645, 734, and 815 nm. The experimental results demonstrated that AIPH decomposed to generate more ABTS^+•^ with increasing temperature (NGL44), as evidenced by the increased UV–visible absorbance (Supplementary Figure 3). The production of alkyl radicals by heating AIPH in nanogels was proven. The release behavior of alkyl radicals from NGL44 using a dialysis bag showed higher radical release from the environment at pH 6.5 than that at pH 4.5 (Figure 1(h)), indicating the capability of free radical release from the nanogels at pH 6.5. The pH values of 4.5 and 6.5 corresponded with the environments of the SC and epidermis, respectively.

### 3.2. Biocompatibility and keratinocyte internalization of the nanogels

The biocompatibility of the nanogels was tested in the presence of a human keratinocyte cell line (HaCaT). The MTT assay demonstrated that NGL was nontoxic to HaCaT cells at HPTS concentrations ranging between 25 and 125 μg/mL (Supplementary Figure 4). The increase in HPTS concentration corresponded to the increased concentration of the nanoparticles. Keratinocyte viability remained >80% even at the highest nanogel concentration, suggesting negligible cytotoxicity. The NGL containing 25 μg/mL HPTS was chosen for further cell uptake study. The capability of keratinocytes to engulf NGL was evaluated by flow cytometry. NGL labeled with fluorescent HPTS served as the fluorescence indicator of cell internalization. The confocal image illustrated the ingestion of HPTS by keratinocytes after nanogel incubation. The internalization of free HPTS (control) was very low (Supplementary Figure 5). The NGL treatment of HaCaT cells resulted in a rightward shift of the peak in the histogram compared with the nontreatment control (Figure 2(a)), indicating NGL uptake by the cells. Quantitative flow cytometry analysis showed that 67% of the HaCaT cells were fluorescent after treatment with NGL (Figure 2(b)). As NGL revealed quite different diameters at pH 5 and 6, the effect of pH on NGL uptake by keratinocytes was also examined (Figure 2(c)). Upon coculture with NGL for 24 h, the pH 6 condition exhibited a rightward shift compared with the pH 5 condition in the flow cytometry results. However, the fluorescence intensity inside the keratinocytes was comparable (Figure 2(d)). This indicated that the nanogel size did not affect endocytosis by keratinocytes. To further investigate the ability of nanogel internalization in keratinocytes, we used DCFDA as the fluorescence indicator to measure the alkyl radical inside the cells. DCFDA is an intracellular probe for free radicals, which functions due to its reaction with radicals to generate dichlorofluorescein with an intense green signal. Confocal images displayed no observable green fluorescence in the NGL-treated group (Figure 2(e)). NGL44 intervention resulted in green fluorescence owing to the residence of alkyl radicals in the nanogel structure after heating. The greater alkyl radical amount of NGL44 in the cells than NGL was confirmed by flow cytometry (Figure 2(f,g)). More alkyl radicals were observed in keratinocytes treated with NGL44 than in the others.

### 3.3. Nanogel-induced keratinocyte death

**Figure 3.**
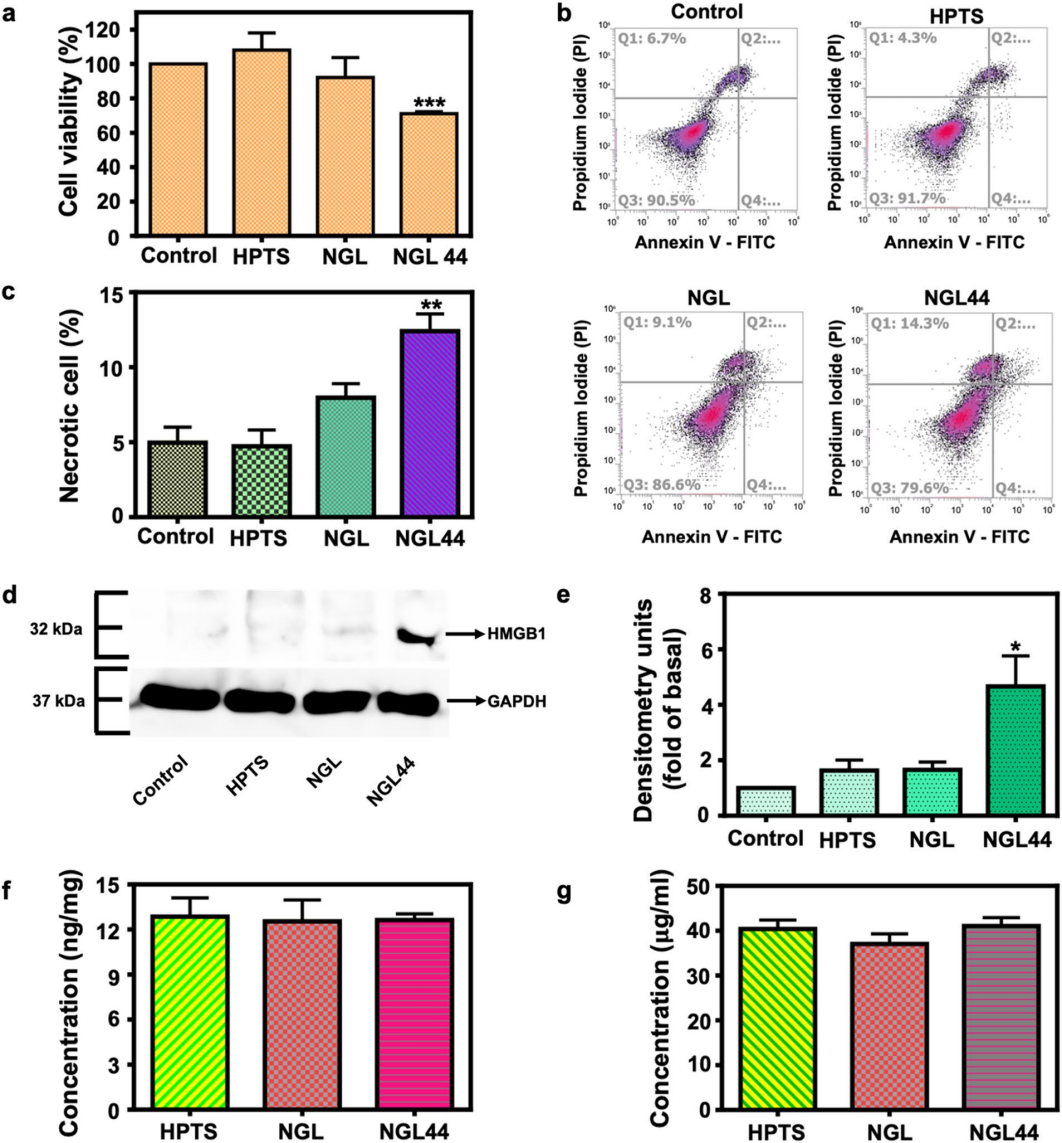
*In vitro* effect of alkyl radicals on keratinocytes and the ability of nanogels to penetrate across intact pig skin: (a) cell viability of human keratinocytes incubated with HPTS, NGL, and NGL44 evaluated using MTT assay; (b, c) cell death mechanism analysis using annexin V and propidium iodide through flow cytometry; (d, e) Western blotting assay of HMGB1 in keratinocytes after treatment with HPTS, NGL, and NGL44; (f) HPTS amount in skin deposition from free form, NGL, and NGL44; (g) HPTS amount in receptor compartment of Franz cell after treatment with free form, NGL, and NGL44. All data are expressed as mean and SEM (*n* = 6). *p < .05, **p < .01 ***p < .001, compared with respective control groups.

An MTT assay was employed to determine whether nanoencapsulated alkyl radicals could cause keratinocyte death to suppress proliferation (Figure 3(a)). Free HPTS and NGL did not exert significant cytotoxicity against HaCaT cells. NGL44 presented a superior cell death effect compared with the other groups. The cell viability was decreased from 91% (NGL) to 70% (NGL44, *p* < 0.001) after nanogel heating, which demonstrated that the conversion of AIPH to alkyl radicals was effective in promoting cytotoxicity. The possible mode of cell death evoked by NGL44 was elucidated by flow cytometry. Standard annexin V/PI costaining was used to examine the HaCaT cell death pathway (Figure 3(b)). NGL44 treatment elicited obvious cell necrosis (Q1). The late and early apoptotic keratinocytes (Q2 and Q4) were not significantly increased after NGL44 intervention compared with the control. A minimum amount of necrosis was detected for the other treatment groups. The quantitative flow cytometry data showed that the necrotic rate of HaCaT cells treated with NGL44 was 12.4%, which was significantly higher than that of HaCaT cells treated with free HPTS and NGL (Figure 3(c)). HMGB1 is a signal of cell necrosis that can be released when the plasma membrane loses integrity. The expression of HMGB1 in HaCaT cells after treatment with NGL and NGL44 was detected by immunoblotting (Figure 3(d)). We observed upregulation of HMGB1 in the NGL44 treatment group, whereas NGL did not change HMGB1 expression compared with the control. NGL44 increased HMGB1 expression by approximately 5-fold compared with the control. (Figure 3(e)). This result verified the ability of NGL44 to induce keratinocyte necrosis. Nanogel delivery into and across pig skin was explored in a Franz diffusion cell. Both skin deposition and receptor accumulation of the nanogels were determined. We used baby pig skin, which is thinner than human skin, as the permeation model. Thus, the deposition of nanogel in skin and the receptor compartment can be regarded as transport into the superficial layer and deeper skin strata, respectively. The skin absorption of the nanogels was calculated based on the fluorescence detection of HPTS. We observed similar skin permeation between the free and nanoencapsulated HPTS in terms of both skin deposition and receptor amount (Figure 3(f,g)). These data signified the possible delivery of nanogels into deeper skin strata, although the nanogels had larger dimensions than free HPTS. The nanogels should at least penetrate into the viable epidermis, which is the target site for proliferated keratinocyte clearance.

### 3.4. In vivo psoriasiform dermatitis study

**Figure 4.**
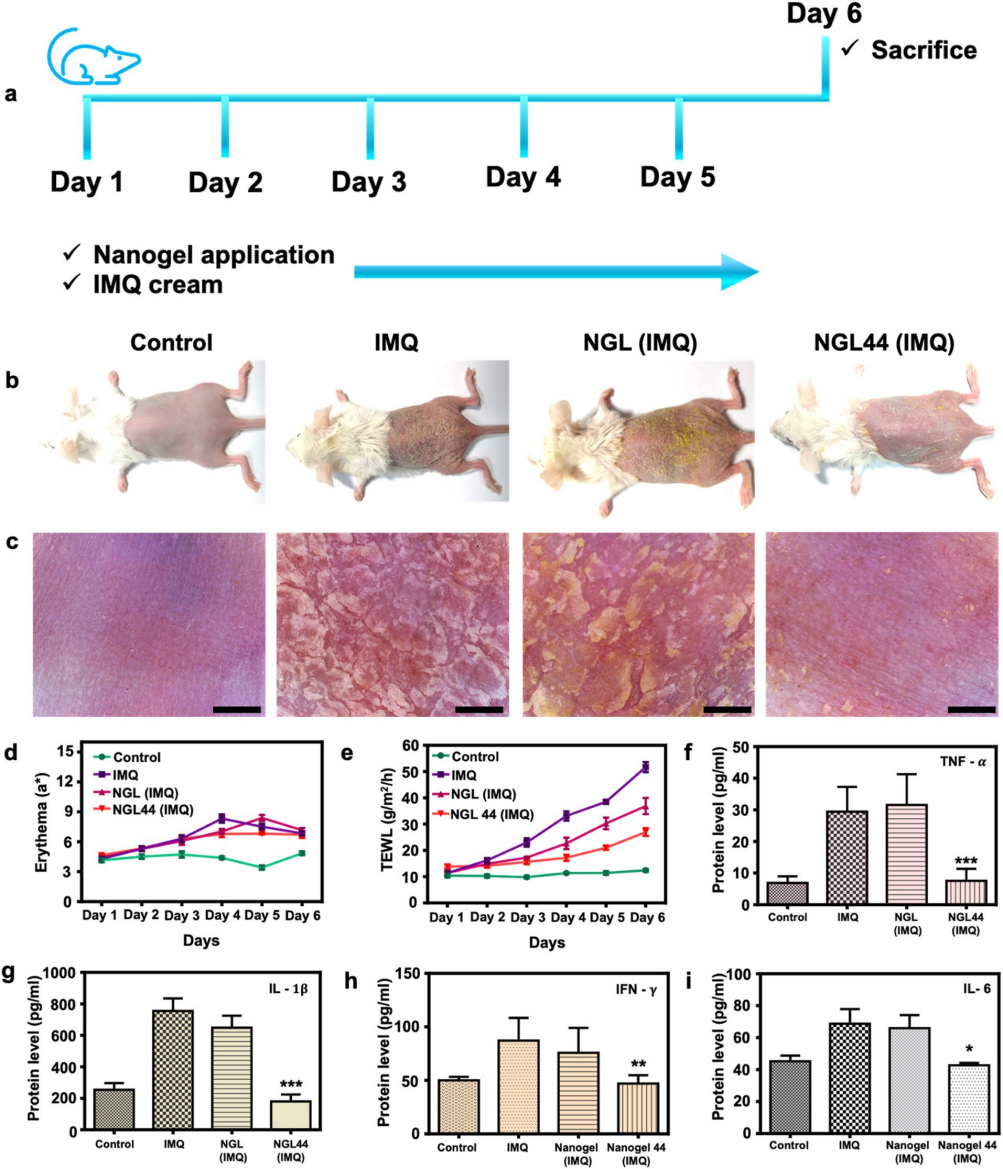
*In vivo* effectiveness of nanogels on psoriasiform lesions in the murine model: (a) schematic representation of *in vivo* experimental procedure on murine model; (b) the gross images of mouse back taken using digital camera on Day 6; (c) the close-up images taken using hand-held digital microscope on Day 6. Scale bar represents 1 μm; (d) Erythema (a*) on mouse skin from Days 1 to 6; (e) TEWL on mouse skin from Days 1 to 6; (f) the expressed protein level of TNF-α in the punched psoriasiform skin; (g) the expressed protein level of IL-1β  in the punched psoriasiform skin; (h) the expressed protein level of IFN-γ in the punched psoriasiform skin; (i) the expressed protein level of IL-6 in the punched psoriasiform skin. All data are expressed as mean and SEM (*n* = 6). *p < .05, **p < .01, ***p < .001, compared with respective control groups.

The therapeutic efficiency of the nanogels against psoriasiform lesions was evaluated in a murine model. IMQ cream activated the mouse skin to create psoriasis-like symptoms. NGL or NGL44 was topically applied to the skin every day for 5 days to understand the ability to inhibit psoriasiform hyperplasia and inflammation (Figure 4(a)). Both macroscopic and microscopic characteristics of the skin surface displayed erythema covered with scales after IMQ treatment (Figure 4(b,c)). Limited amelioration of these signs was observed in the NGL treatment group. Redness and plaque were significantly arrested in the NGL44 group only. A few scabs were still present after a 5-day application of NGL44. Topical NGL44 largely decreased the severity score of PASI from 11 to 1 compared with IMQ intervention alone (Supplementary Figure 6). Erythema quantified by colorimetry (a*) showed a significant increase with IMQ cream intervention (Figure 4(d)). NGL44 moderately lessened the erythema from Day 4. As an indicator of skin barrier function and psoriasis severity, TEWL increased from 12.4 to 51.7 g/m^2^/h after 5 days of IMQ activation (Figure 4€). This indicated the dysfunctional barrier property of psoriasiform skin. The elevated TEWL induced by IMQ was decreased to 36.9 and 27.0 g/m^2^/h after NGL and NGL44 were applied for five consecutive days. The expression of cytokines in the skin was evaluated after 5-day treatment with IMQ and nanogels. Upon activation by IMQ, the expression of TNF-α, IL-1β, IFN-γ, and IL-6 was increased by 4-, 3-, 2-, and 1.5-fold compared with the normal control (Figure 4(f–i)). NGL was unable to suppress the upregulation of cytokines induced by IMQ. These proinflammatory mediators in psoriasiform skin were significantly decreased by topically applied NGL44. This heated nanogel generally recovered the cytokine level to the baseline control.

**Figure 5.**
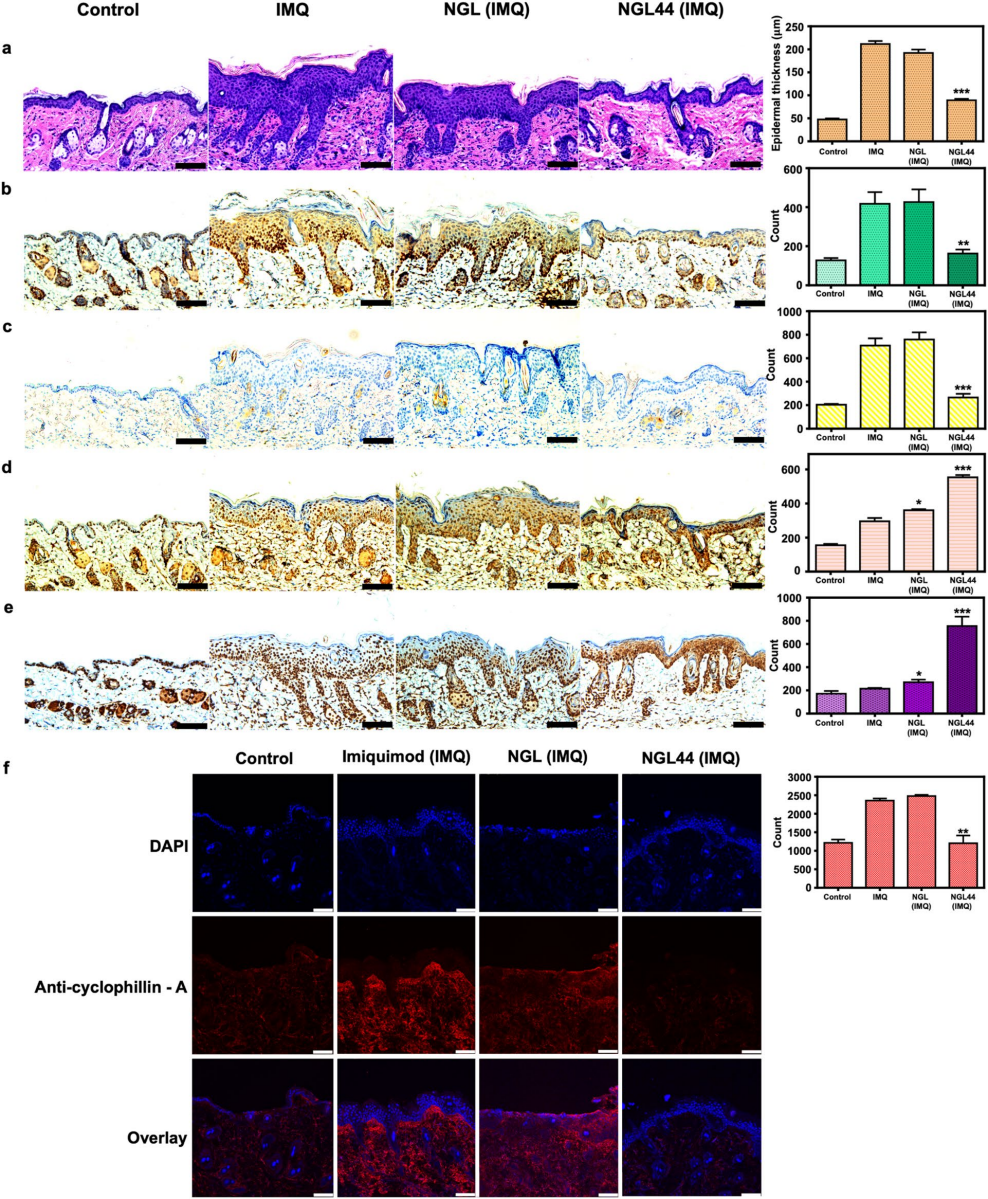
Immunohistochemical analysis of the psoriasiform lesions after treatment of the nanogels: (a) H&E staining; (b) Ki67; (c) Ly6G; (d) 8-OHDG; (e) HMGB1; (f) Cyclophilin A. Scale bar represents 100 μm for (a–e) and 75 μm for (f). All data are expressed as mean and SEM (*n* = 6). **p < .01 ***p < .001, compared with respective control groups. SEM: scanning electron microscopy.

The H&E-stained histology represented healthy skin with an intact SC and organized thin epidermis (Figure 5(a)). IMQ intervention led to desquamation, hyperkeratosis, and acanthosis along with elongated rete ridges. IMQ-stimulated skin also depicted distinct signs of immune cell recruitment in the dermis. NGL did not significantly improve these signs of psoriasiform hyperplasia and inflammation. NGL44 could smooth and thin the psoriasiform skin. The infiltrating inflammatory cells in the plaque were also mitigated by NGL44. Treatment with NGL44 led to a reduction in psoriasiform epidermis thickness from 211.5 to 89.2 μm, suggesting inhibition of epidermal thickening. Ki67 is a proliferation biomarker. This proliferation indicator was largely found (3-fold) in the basal and suprabasal layers of the epidermis after IMQ activation (Figure 5(b)). NGL-treated Ki67 IHC was similar to that of IMQ treatment alone. On the other hand, NGL44 decreased the number of Ki67-positive cells by 61%. Neutrophil infiltration in skin is a hallmark of psoriasis. Neutrophil accumulation in the dermis was observed by Ly6G IHC (Figure 5(c)). Brown staining in the dermis indicated increased Ly6G distribution after IMQ stimulation. This infiltration was restrained after topical delivery of NGL44. As an oxidative stress indicator, 8-OHDG expression was increased in IMQ-treated skin compared with healthy control skin (Figure 5(d)). This is reasonable, as the development of psoriasis can elevate oxidative stress in the skin. NGL slightly but significantly increased 8-OHDG in psoriasiform lesions according to the quantitative analysis. A further increase in 8-OHDG was visualized in the NGL44 treatment group. The necrotic cells were counted by HMGB1 IHC (Figure 5(e)). IHC demonstrated HMGB1 expression in the stratum basale of the psoriasiform skin. Intracellular necrosis is recognized during the evolution of psoriasis. We found clouds of HMGB1 expression in the NGL44-treated psoriasiform epidermis. HMGB1 was detectable in both intracellular and extracellular areas. HMGB1 may have leaked out from the permeabilized necrotic cells because of the cellular stress prompted by nanoencapsulated alkyl radicals. Cyclophilin A is a proinflammatory factor that boosts inflammation through IL-1β upregulation. The overexpressed cyclophilin A distributed in IMQ-stimulated skin was diminished by topical NGL44 but not NGL (Figure 5(f)).

### 3.5. In vivo skin absorption of nanogels

**Figure 6.**
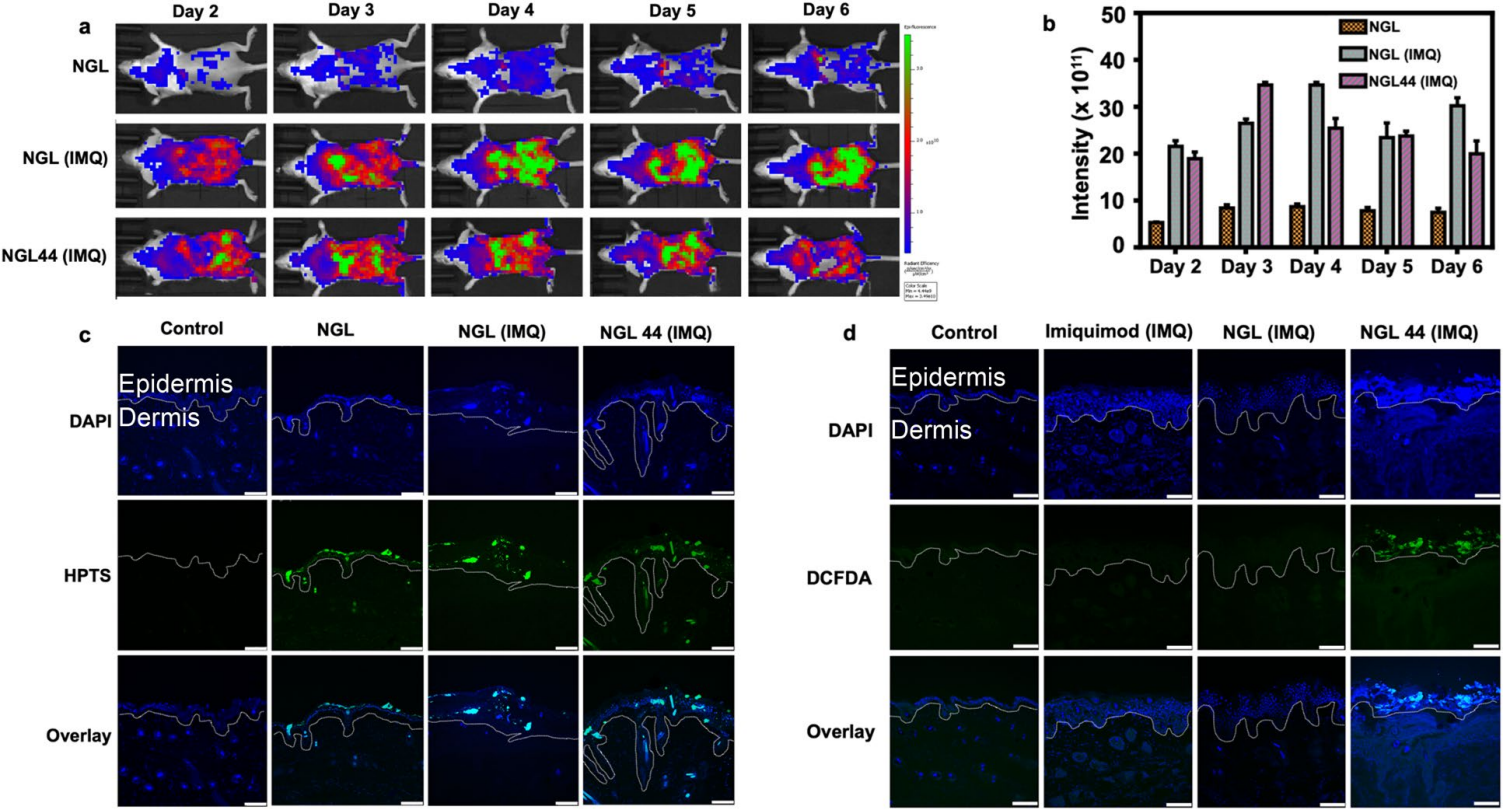
Chitosan nanogels for delivering alkyl radical to mouse skin: (a) topical visualization of NGL and NGL44 presence on mouse back skin employing IVIS in healthy and psoriasiform models; (b) the fluorescence intensity measured from IVIS; (c) confocal visualization of nanogels on the dissected skin section; (d) confocal visualization of alkyl radical on the dissected skin section. Scale bar represents 75 μm. The white lines indicate the epidermal–dermal junction in (c) and (d). All data are expressed as mean and SEM (*n* = 6). IVIS: *in vivo* imaging system; SEM: scanning electron microscopy.

HPTS is a pH-sensitive fluorescence dye that can be considered to reflect the severity of psoriasis because of the pH change in psoriatic lesions. We showed that the skin surface pH values of healthy and psoriasiform mice were 4.9 ± 0.3 and 4.4 ± 0.1, respectively. This suggested the acidification of the skin surface in psoriasis development. *In vivo* bioimaging of the skin after HPTS-loaded nanogel application was performed by IVIS. Imaging was captured after the treatment of nanogels each day from Day 2 to Day 6 post-IMQ intervention. Compared with healthy skin, NGL revealed more intense fluorescence in psoriatic lesions (Figure 6(a,b)). There was no autofluorescence in the mouse back skin without treatment (Supplementary Figure 7). This suggested that the fluorescence signal from the nanogel-treated skin could be due to nanogel absorption. This result suggested increased HPTS fluorescence with decreasing skin pH. NGL44 generally displayed weaker fluorescence intensity than NGL. This could be due to the partial recovery of the psoriasiform lesion to the normal condition after NGL44 treatment. Ex vivo bioimaging of the skin excised at Day 6 was conducted to examine the nanogel distribution. Application of the nanogels on healthy and psoriasiform skin samples resulted in apparent nanogel delivery into the viable epidermis (Figure 6(c)). Some fluorescence was detectable in the dermis. No significant difference in the absorption amount or distribution was found between NGL and NGL44. DCFDA was utilized to observe the free radical distribution in skin (Figure 6(d)). No green fluorescence from DCFDA was observed in the NGL-treated skin, indicating a limited amount of alkyl radicals or cellular stress in the skin. Compared with the NGL group, significant fluorescence was observed in the psoriasiform lesion of the NGL44 group. This nanogel generated cellular stress principally in the epidermis.

### 3.6. Safety of nanogels on mouse skin

**Figure 7.**
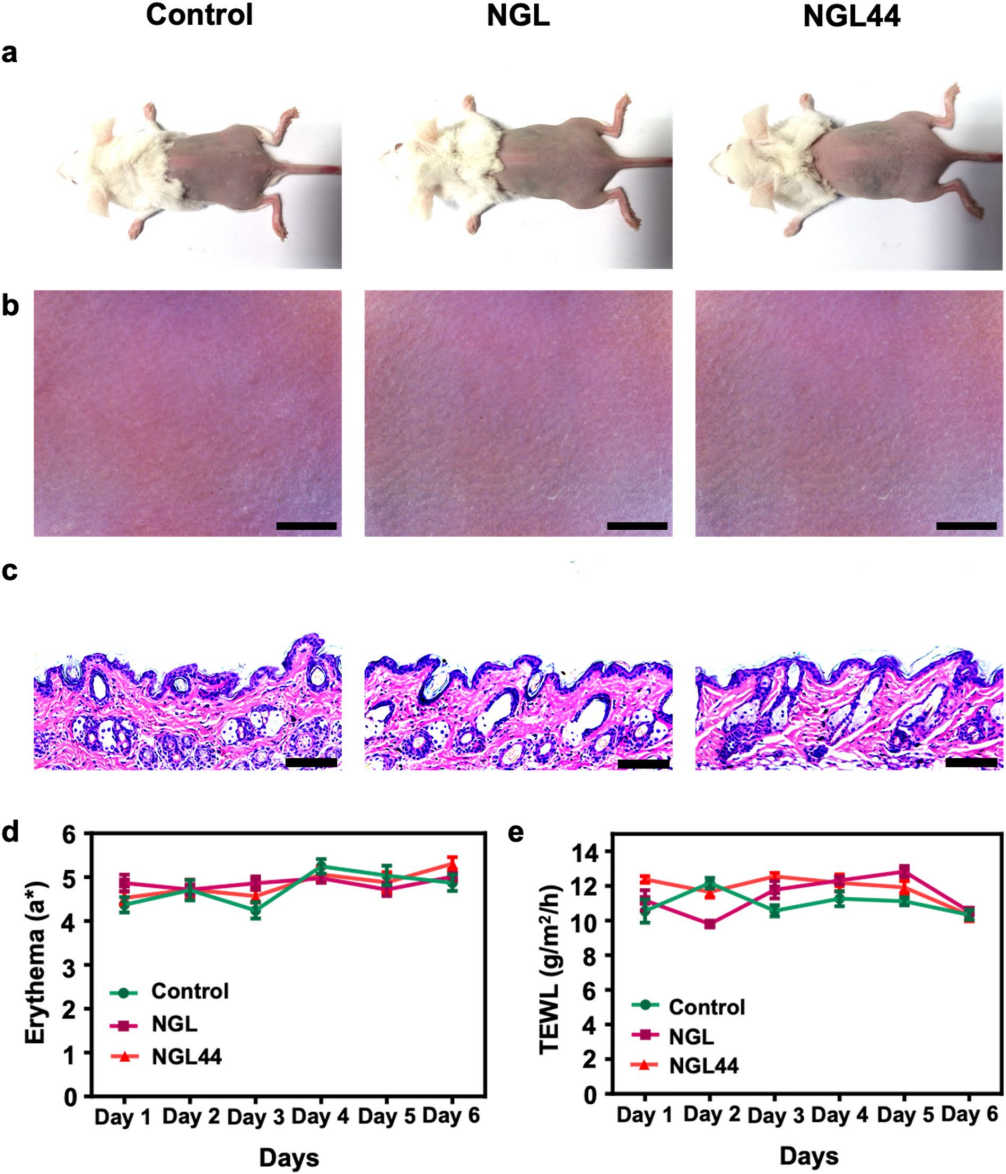
*In vivo* safety of the topically applied nanogels (200 μl) after a 5-day consecutive administration on mouse back skin: (a) the gross image of mouse back skin taken using a digital camera; (b) the digital microscopic image of mouse back skin; (c) H&E staining; (d) erythema (a*) on back skin; (e) TEWL on back skin. All data are expressed as mean and SEM (*n* = 6). SEM: scanning electron microscopy; TEWL: transepidermal water loss.

An ideal skin delivery formulation should accomplish the therapeutic goal without inflicting cutaneous irritation. Having established the theranostic potential of the nanogels, we next considered the *in vivo* tolerability of NGL and NGL44. Skin irritation was assessed using a repeat application of the nanoformulations for 5 days. Visual observation showed no signs of skin irritation, such as redness and swelling, after nanogel treatment compared with the saline control (Figure 7(a,b)). Histological examination revealed a normal and distinct epidermal layer after nanogel application (Figure 7(c)). When NGL and NGL44 were applied to healthy skin, the dermal layer was free of edema and inflammation. Quantitative erythema (a*) also showed a comparable level between the control and nanogel treatment groups (Figure 7(d)), suggesting no evidence of inflammation after nanosystem intervention. The same tendency was noted in the TEWL (Figure 7(e)), demonstrating the maintenance of barrier function after nanogel treatment.

## 4. Discussion

Nanogels comprise polymers with crosslinked networks. The pores inside the nanogel structure and the specific charge of the polymers result in the high encapsulation of bioactive compounds or drugs. Polymeric nanogels, as drug nanocarriers, present the advantages of stimuli responsiveness, enhanced biomembrane penetration, increased bioavailability, and reduced adverse effects (Kousalová & Etrych, 2018). In the present study, pH-sensitive chitosan nanogels were prepared to load AIPH and HPTS as topical theranostics for treating psoriasiform hyperplasia. The nanogels could be heated before application to decompose AIPH to produce alkyl radicals, which are responsible for eliciting proliferated keratinocyte death. The swelling–deswelling of the nanogels could be controlled by varying the pH. The designed nanogels were of small size when passing across the SC. After delivery to the viable epidermis with a higher pH environment, the nanogels swelled to release alkyl radicals, inhibiting hyperplasia. Both *in vitro* and *in vivo* experiments demonstrated successful inhibition of keratinocyte proliferation and epidermal hyperplasia by the nanogels. The nanoformulations were also favorable for detecting psoriasis severity because of the pH responsiveness of HPTS.

Nanogels are more responsive to stimuli such as pH than other nanoparticles because of their unique 3D network, which easily changes in different environments (Smeets & Hoare, 2013). The large difference in the nanogel size between hydrodynamic detection and the dry state could verify this perspective. The flexibility in nanogel size in responding to external stimuli makes nanogels an ideal nanocarrier system for controlling drug delivery. We found that the nanogels showed swelling at pH 6 and 7.4, whereas a deswelling pattern was detectable in the acidic environment (<pH 5.5). The reduction in chitosan nanogel size under acidic conditions has been demonstrated previously (Qi et al., 2016), and it is due to the disassembly of the particles. The increased chitosan solubility in acidic solution leads to protonation of the amino moiety. This protonation ensures chain relaxation, resulting in rapid hydrogen bond dissociation. The protonation extent is reduced when the pH is increased (Algharib et al., 2020). The decreased zeta potential at pH 6 compared with that at pH 5 confirmed this concept. The reduced protonation led to the agglomeration of nanogels. This phase transition also allowed nanogel swelling due to water penetration into the free space of the nanostructure. In the deswelling state, we expected that the nanogels could protect AIPH or free radicals from release due to their rigid structure with less water. This reduced dimension of nanogels permits nanogel transport across biological barriers (Cuggino et al., 2019). Our ABTS assay manifested a diffusional release of alkyl radicals from the nanogels, indicating possible swelling in response to pH to accelerate free radical escape from the crosslinked network.

The cellular uptake results indicated that the nanogels are ideal candidates for intracellular delivery. This nanogel internalization in keratinocytes was evidenced by flow cytometry and confocal microscopy. Positively charged chitosan nanogels prefer nonspecific binding to the cell membrane through electrostatic interactions, resulting in facile endocytosis (Schütz et al., 2011; Algharib et al., 2020). Although the nanogels at pH 6 exhibited larger sizes than those at pH 5, the cellular uptake between both pH conditions was approximately equal. The level of nanogel internalization by HaCaT cells was determined by estimating the HPTS fluorescence inside the cells. As free HPTS shows very low permeability into the cells (Cao et al., 2021), we hypothesized that only the nanogel form could carry HPTS into the cells. This meant that the large nanogels (at pH 6) could still be engulfed by keratinocytes. The hydrodynamic diameter of the nanogels at pH 6 was determined to be approximately 8000 nm after a 100-fold dilution with water. We expected that the actual nanogel size in the cell culture medium was relatively small compared with the hydrodynamic size because of the presence of cells, serum, glucose, and inorganic salts other than water.

Intracellular nanogel delivery led to the higher bioavailability of the therapeutic agent in keratinocytes. The inhibition of keratinocyte proliferation is a critical factor for alleviating psoriasis. We found that the cellular uptake of the nanogels induced the necrosis of keratinocytes. This effect was observed only in the case of NGL44 due to the production of alkyl radicals from AIPH decomposition. Keratinocyte death only occurred in the presence of alkyl radicals and not AIPH. Our DCFDA assay showed an accumulation of alkyl radicals inside the cells after NGL44 treatment. It was expected that nanogel heating could convert AIPH to alkyl radicals in the nanoparticles. Free radicals provoke lipid peroxidation, protein damage, and DNA breakage, contributing to cell death in the process of necrosis and/or apoptosis (Chiang et al., 2017). Cell membrane disruption and disintegration can cause necrosis. Cell membrane damage is one of the mechanisms of action of UV phototherapy against psoriasis (Kemény et al., 2019). Alkyl radicals can trigger cellular stress via the necrosis/apoptosis pathway in both normoxic and hypoxic environments. Under normoxic conditions, alkyl radicals convert into alkoxyl radicals in the presence of cellular oxygen to induce oxidative stress and ultimately lead to cell death (Wang et al., 2017a). In hypoxia, alkyl radicals cause direct DNA damage and glutathione depletion to accumulate oxidative stress in cells (Liang et al., 2022). In addition to nanogel internalization into keratinocytes to trigger necrosis, we hypothesized that the free alkyl radicals released from the nanogels could also play a role in causing cell death. The dialysis bag study validated the possible release of alkyl radicals from the particles. Both nanoencapsulated and free alkyl radicals were responsible for nanogel-induced necrosis.

Keratinocytes can be considered as the resident immune system in the skin. The hyperproliferation of keratinocytes due to inflammatory immune stimulation is the fundamental feature of psoriasis (Zhou et al., 2022). The scaly plaque in psoriasis highlights epidermal hyperproliferation (Pohla et al., 2020). Hyperproliferation is also reflected by the histological characteristics of hyperkeratosis and acanthosis. Our psoriasiform animal study demonstrated the effective inhibition of epidermal thickening by heated nanogels. The IHC analysis for Ki67 provided evidence of this effect of NGL44. A prerequisite for achieving antipsoriatic activity by the nanogels was that the nanoparticles should penetrate into the skin and deliver the active ingredients to the target region. A greater permeation of free HPTS than the nanogel form was anticipated because the free molecules exhibited a smaller size than the nanoparticles. Nevertheless, the nanogel-associated HPTS showed skin absorption comparable with that of the free form. This suggested moderate delivery of nanogels into the skin. The deteriorated barrier function of psoriasiform skin leads to the possible transport of nanoparticles into the skin (Lee et al., 2022). The imperfect SC structure of psoriasiform skin might allow the direct entrance of the nanoparticles to reach viable epidermis. It is inferred that the nanogel size was largely increased in viable skin with a higher pH than in SC. The nanoparticle aggregates were not easy to transport into the Franz cell receptor at pH 7.4. HPTS accumulation in the receptor compartment could be expected in the form of free HPTS molecules but not nanoparticulate HPTS.

Chitosan nanogels offer the advantages of combined bioadhesive properties and a large surface area to increase the contact time with the skin surface, which makes them an ideal topical delivery system (Chellappan et al., 2019). The positive charge of the nanogels is crucial for interacting with the negatively charged SC and disorganizing the tight junctions in the epidermis to facilitate permeation (Elkomy et al., 2022). The softness of the nanogels leads to possible deformability when diffusing into the SC (Scotti et al., 2022). After transport across the SC, the high water percentage and hydrophilic nature of chitosan nanogels can increase their retention in the hydrophilic epidermis (Islam et al., 2017). Alkyl radicals should be delivered to the deep epidermis where hyperproliferation occurs. *In vivo* skin absorption substantiates significant accumulation of nanogels in the epidermal layer. The nanogels showed promising results in assisting active ingredient delivery to the epidermis. After distribution in the epidermis, the nanogels were internalized by keratinocytes to trigger cell death. Another possibility was the release of alkyl radicals from the nanogels before internalization. The release could be limited in the SC (pH 4.0–4.5) because of the deswelling state of the nanogels. After entering the epidermis with higher pH, a pH-triggered rapid release occurred in the aggregation and swelling state. *In vivo* DCFDA imaging demonstrated the generation of oxidative stress mainly in the epidermis after topical delivery of NGL44. The *in vivo* animal study established proof of principle that the destruction of proliferative keratinocytes was adequate to clear the plaque and psoriasiform inflammation.

HMGB1 IHC validated that necrosis was the primary pathway of nanogel-induced keratinocyte death. We observed possible movement of HMGB1 outside the cells in the epidermis. One feature of necrosis is the loss of cell membrane integrity and subsequent intracellular HMGB1 escape (Magna & Pisetsky, 2014). HMGB1 is reported to attenuate skin inflammation by inducing the accumulation of platelet-derived growth factor receptors (Aikawa et al., 2015). Hence, the inhibition of epidermal thickening could lead to decreased psoriasiform inflammation. Psoriatic lesions are a result of dysregulated interactions between immune cells and keratinocytes. The increased keratinocyte proliferation that drives local inflammation is the major cause of the psoriatic phenotype. Once the immune system is activated, immune cells produce proinflammatory mediators to stimulate keratinocyte proliferation. Activated keratinocytes release TNF-α, IL-1β, and IL-6 as chemoattractants for infiltrating immune cells (Griffiths et al., 2021). This is a vicious feedback loop. In addition to keratinocytes, macrophages, neutrophils, lymphocytes, and mast cells produce high levels of cytokines such as TNF-α, IL-1β, and IL-6 to maintain the positive feedback cycle in psoriasis (Nirmal et al., 2021). Our data illustrated that topical NGL44 suppressed the overexpression of these cytokines in the psoriasiform area. The amplification loop of the interplay between immune cells and keratinocytes could be blocked. Cyclophilin A is considered an upstream proinflammatory factor that regulates IL-1β-mediated inflammatory reactions (Yang et al., 2022). We also observed a significant reduction in cyclophilin A in psoriasiform skin upon NGL44 administration. T-cell-derived IFN-γ is abundantly detected in psoriatic lesions (Albanesi et al., 2018). A primary target of IFN-γ is epidermal keratinocytes, which express high levels of IFN-γ receptors. IFN-γ exposure activates aberrant keratinocyte hyperproliferation (Gu et al., 2022). The potent suppression of IFN-γ by NGL44 was favorable for mitigating psoriatic plaques. In the early phase of psoriasis development, TNF-α and IL-1β facilitate the cutaneous accumulation of circulating neutrophils containing abundant TNF and IL-1 receptors (Németh et al., 2020). Our Ly6G IHC of IMQ-activated skin demonstrated neutrophil recruitment in the dermis. The infiltrated neutrophils release elastase, TNF-α, and IL-6 to enhance keratinocyte hyperproliferation (Nakabo et al., 2022). This crosstalk between neutrophils and keratinocytes was shown to be prevented by the nanogels.

Bioimaging has been devoted to clinical practice to help diagnose disease severity (Tuguntaev et al., 2022). The pH-sensitive fluorescence dye can be used to monitor psoriasis severity because of the pH change in psoriatic lesions. The skin pH of psoriatic plaque is usually lower than that of healthy skin (Lin et al., 2015). The present study detected the skin surface pH values of healthy and IMQ-treated mice as 4.9 and 4.4, respectively. The pH-sensitive characteristic of HPTS demonstrated a stronger fluorescence signal in psoriasiform skin than in normal skin after topical administration of the chitosan nanogels. The mitigation of psoriasiform hyperproliferation by NGL44 resulted in attenuated fluorescence in IVIS. The more acidic condition might strengthen the HPTS fluorescence in skin. Due to their bioadhesive properties, chitosan nanogels might stick to the skin surface for a long time. The scales of psoriasiform skin further retained the nanogels on the cutaneous surface without movement. This led to stronger fluorescence of the IMQ-treated skin than normal skin after nanogel application. The fewer scales in the NGL44-treated psoriasiform skin displayed less fluorescence intensity compared with the NGL-treated skin. Another explanation was the possible correlation between the fluorescence intensity and nanogel absorption. For instance, the greater absorption of NGL delivery into psoriasiform skin compared with intact skin resulted in greater fluorescence in the diseased skin in IVIS. Chitosan nanosystems are basically regarded as safe for skin delivery because of their biocompatibility and biodegradability (Ferreira et al., 2022). Through *in vitro* and *in vivo* tests, the chitosan nanogels developed in this study were found to have antipsoriatic potency without detectable skin irritation upon topical treatment.

## 5. Conclusions

We developed dual AIPH- and HPTS-loaded chitosan nanogels for simultaneous therapy and diagnosis in the treatment of psoriasiform hyperplasia. The topical administration of the nanogels was ideal for improving patient compliance due to the convenient application and noninvasiveness. AIPH was decomposed to alkyl radicals after nanogel heating at 44 °C. The pH-responsible nanogels might be capable of transport into the psoriasiform skin and then swelled in viable epidermis to deliver alkyl radicals to cells and/or release them at the desired target. The fast alkyl radical release from the nanogels effectively caused keratinocyte death through the necrosis pathway. The oxidative stress generated by alkyl radicals was significantly elevated inside keratinocytes treated with the nanogels. The topical nanogels could penetrate the epidermis to target proliferative keratinocytes. *In vivo* topical nanogel treatment induced a robust necross effect to relieve epidermal thickening and inflammation in an IMQ-stimulated animal model. Image-guided therapy using chitosan nanogels was useful for monitoring psoriasiform severity. The experimental data in the investigation demonstrated that the nanogels are attractive carriers for the topical permeation of alkyl radicals in psoriasis, as they could deliver the active ingredient at the deeper epidermis necessary to induce cell death without producing skin irritation.

## Supplementary Material

Supplemental Material

## Data Availability

Data available on request from the authors.
