# Supplementary material for: Topically applied pH-responsive nanogels for alkyl radical-based therapy against psoriasiform hyperplasia
Source: Drug Deliv. 2023 Aug 10;30(1):2245169. doi: 10.1080/10717544.2023.2245169 (PMC10416745; doi:10.1080/10717544.2023.2245169)
Supplement: Supplemental Material [file IDRD_A_2245169_SM5295.pptx]

## Slide 1
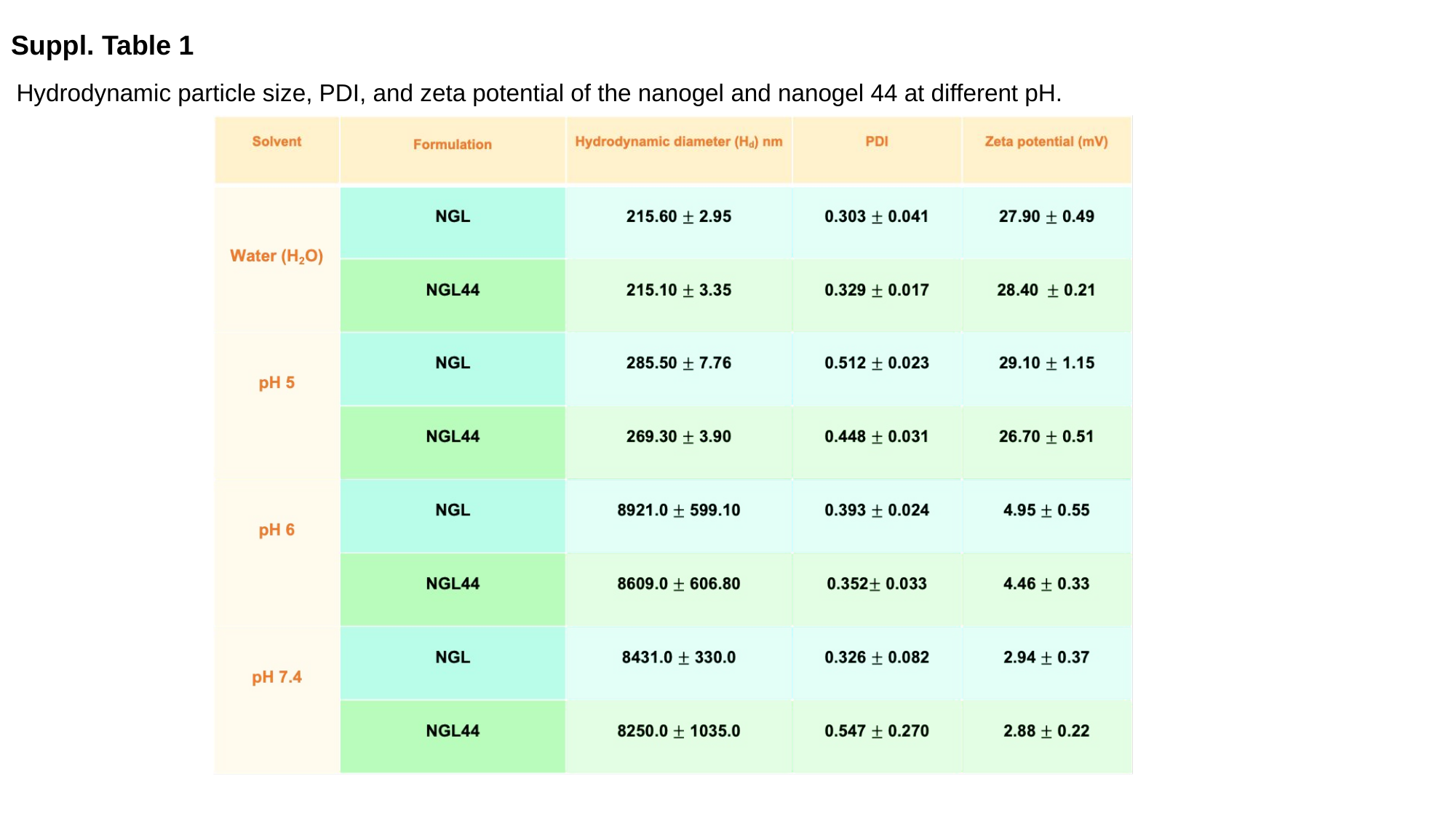

# Suppl. Table 1
Hydrodynamic particle size, PDI, and zeta potential of the nanogel and nanogel 44 at different pH.

## Slide 2
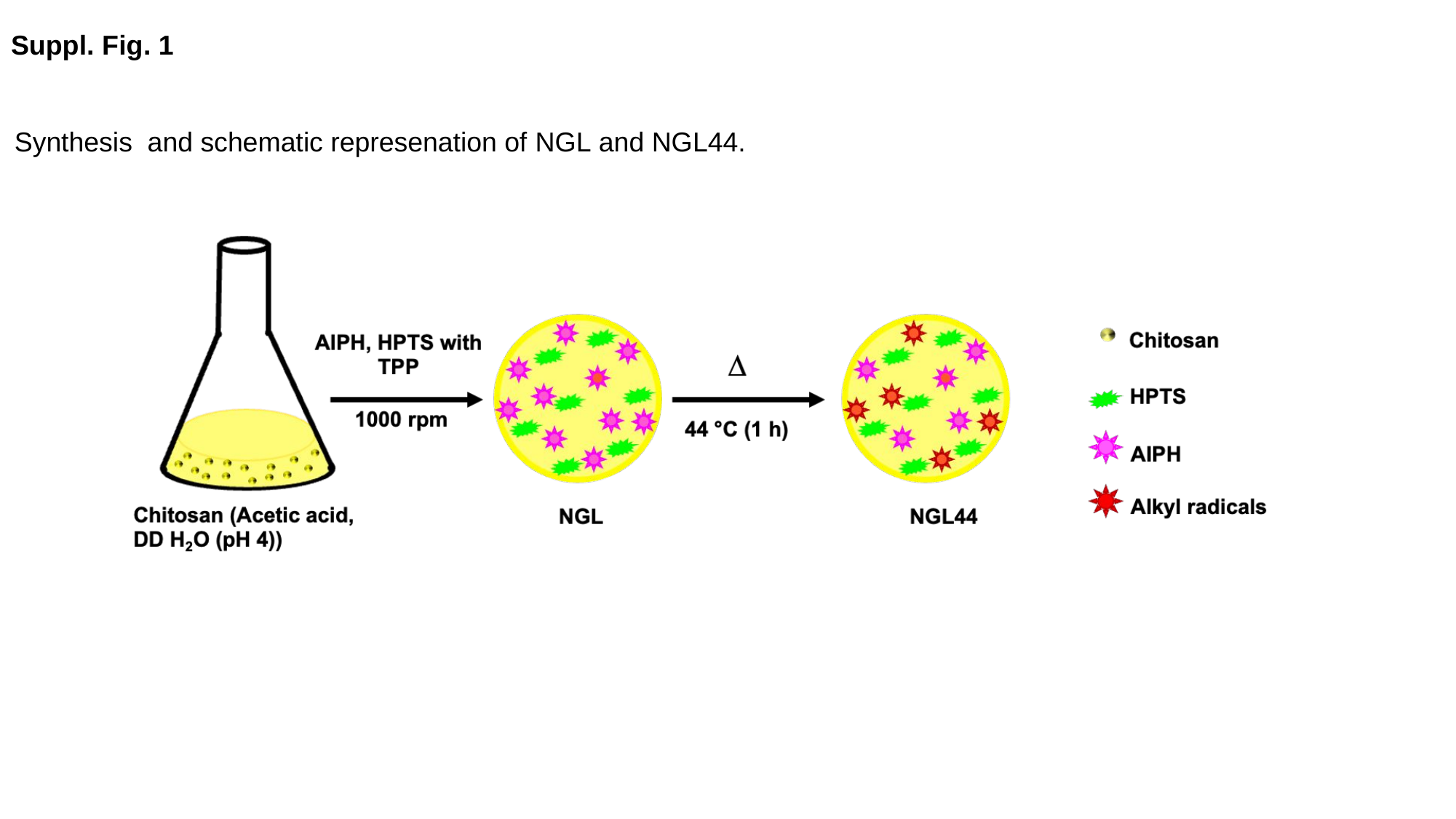

# Suppl. Fig. 1
Synthesis and schematic represenation of NGL and NGL44.

## Slide 3
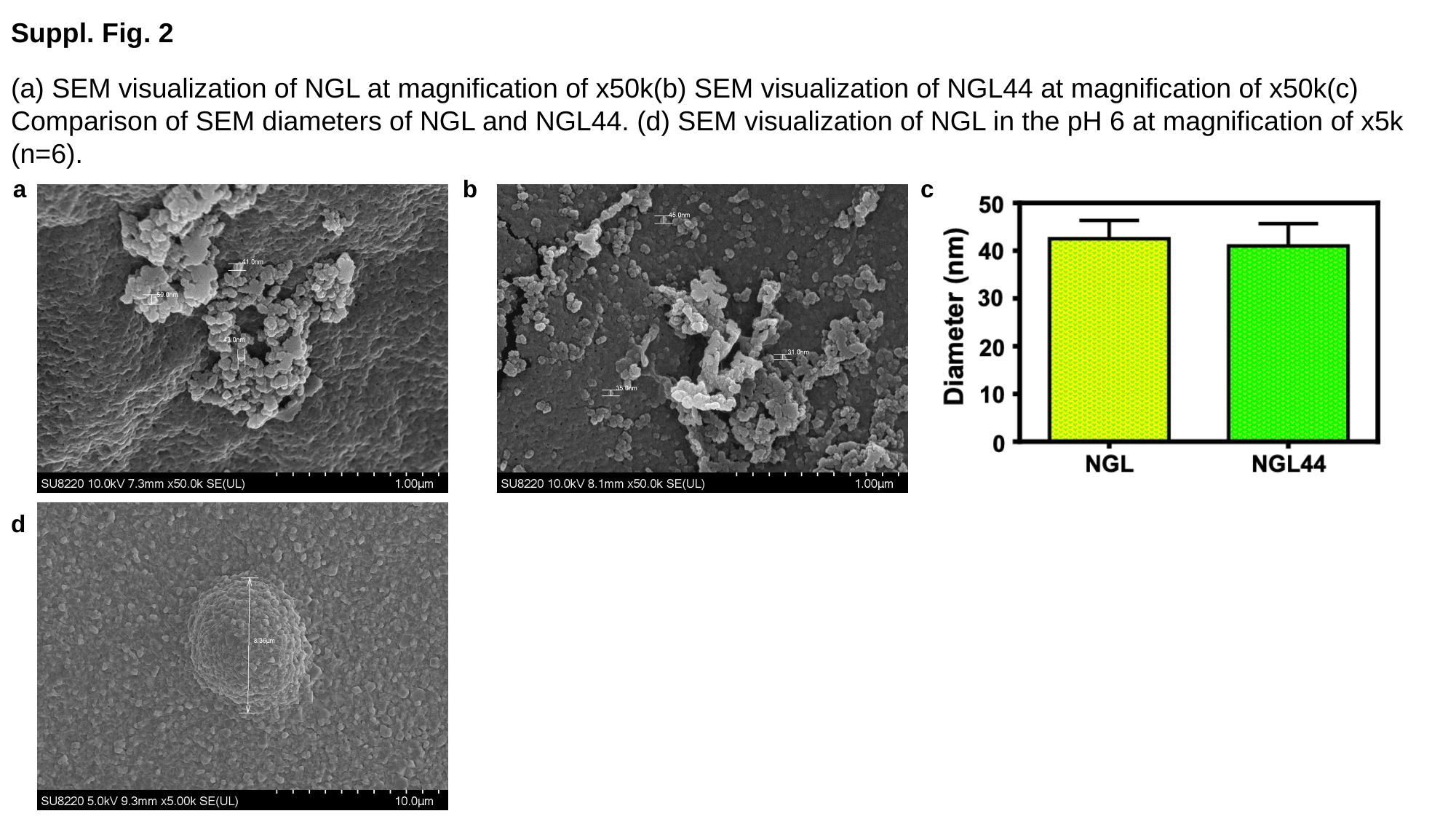

# Suppl. Fig. 2
a
b
c
d

## Slide 4
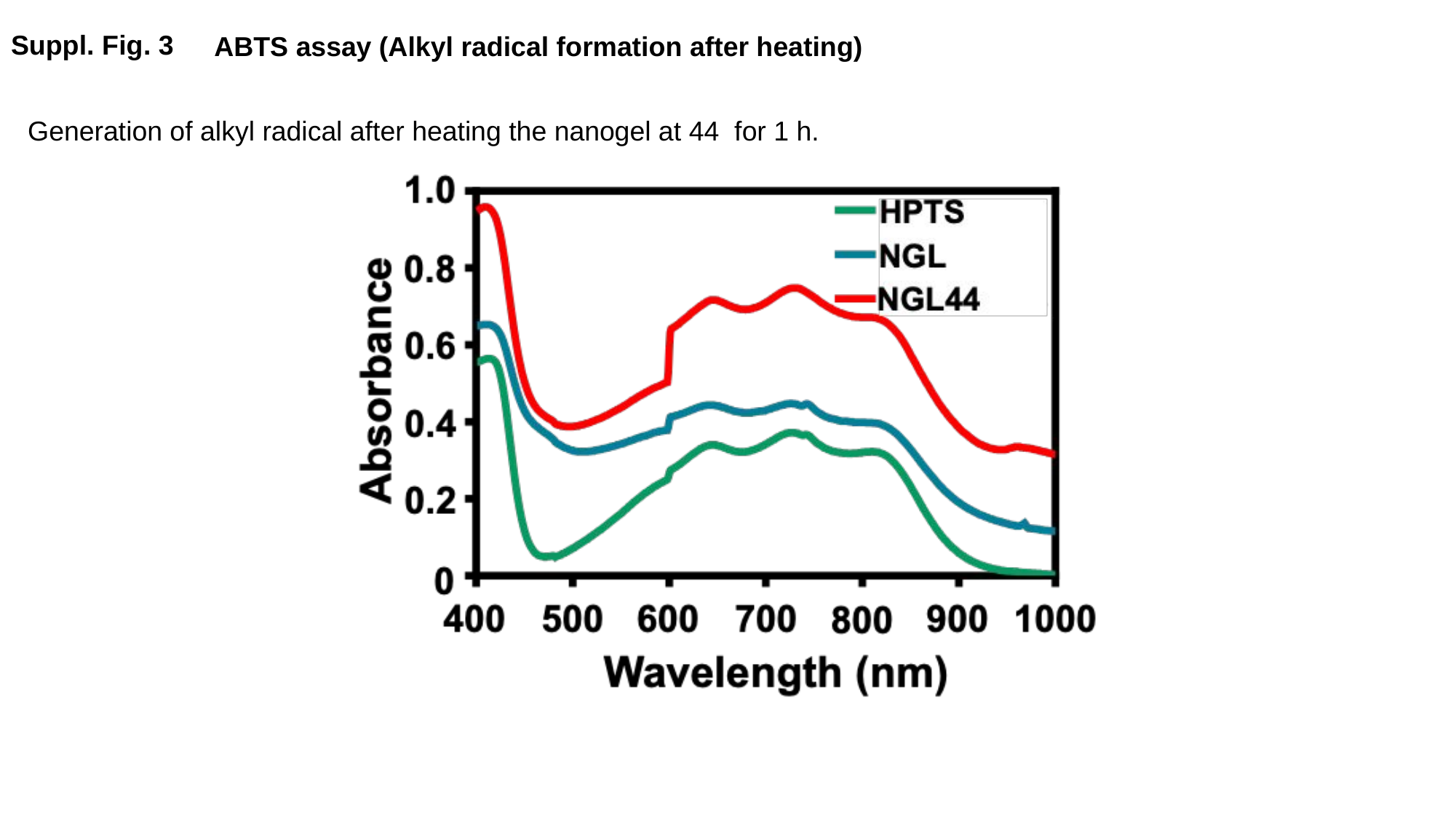

# Suppl. Fig. 3
ABTS assay (Alkyl radical formation after heating)

## Slide 5
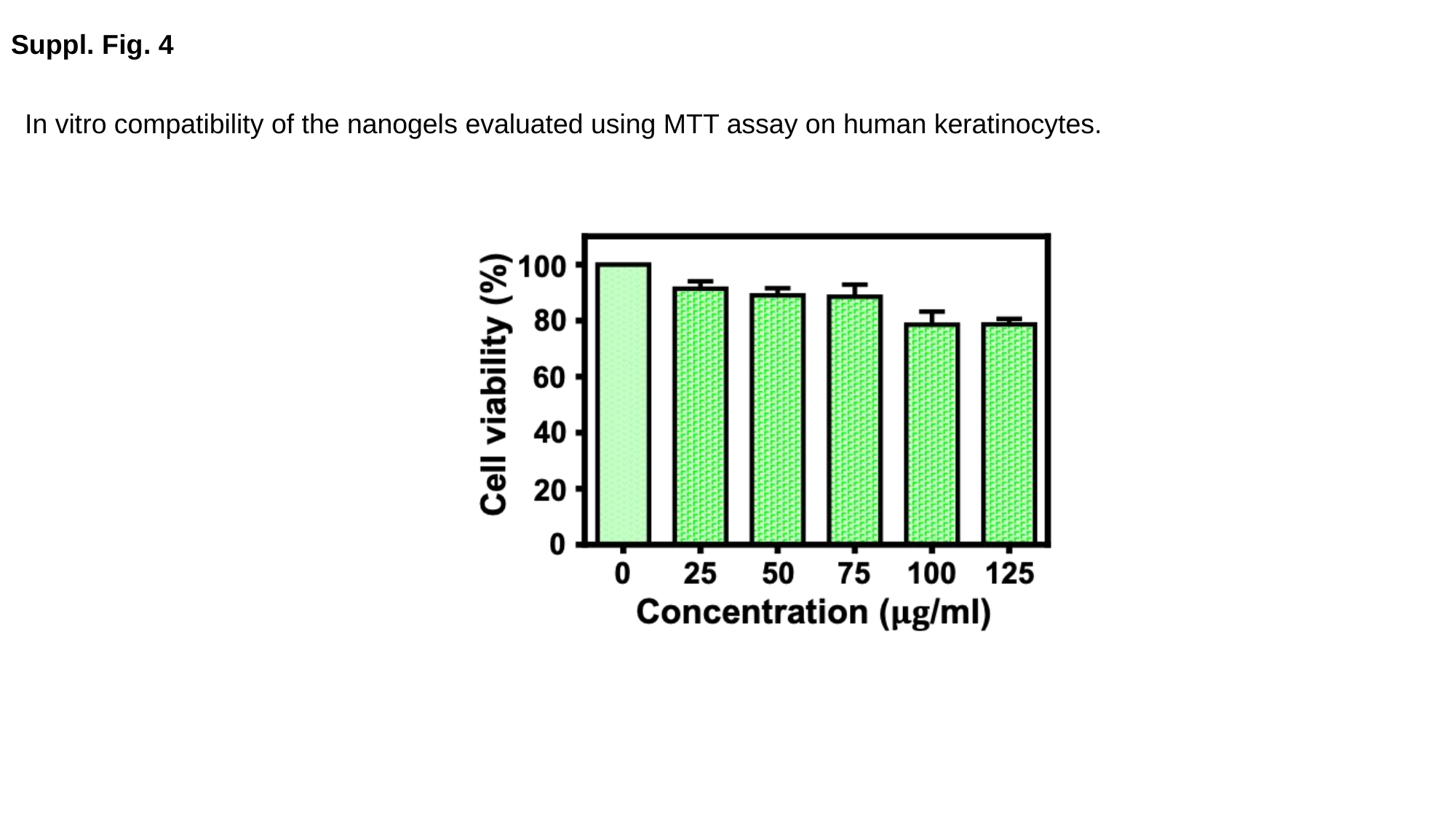

# Suppl. Fig. 4
In vitro compatibility of the nanogels evaluated using MTT assay on human keratinocytes.

## Slide 6
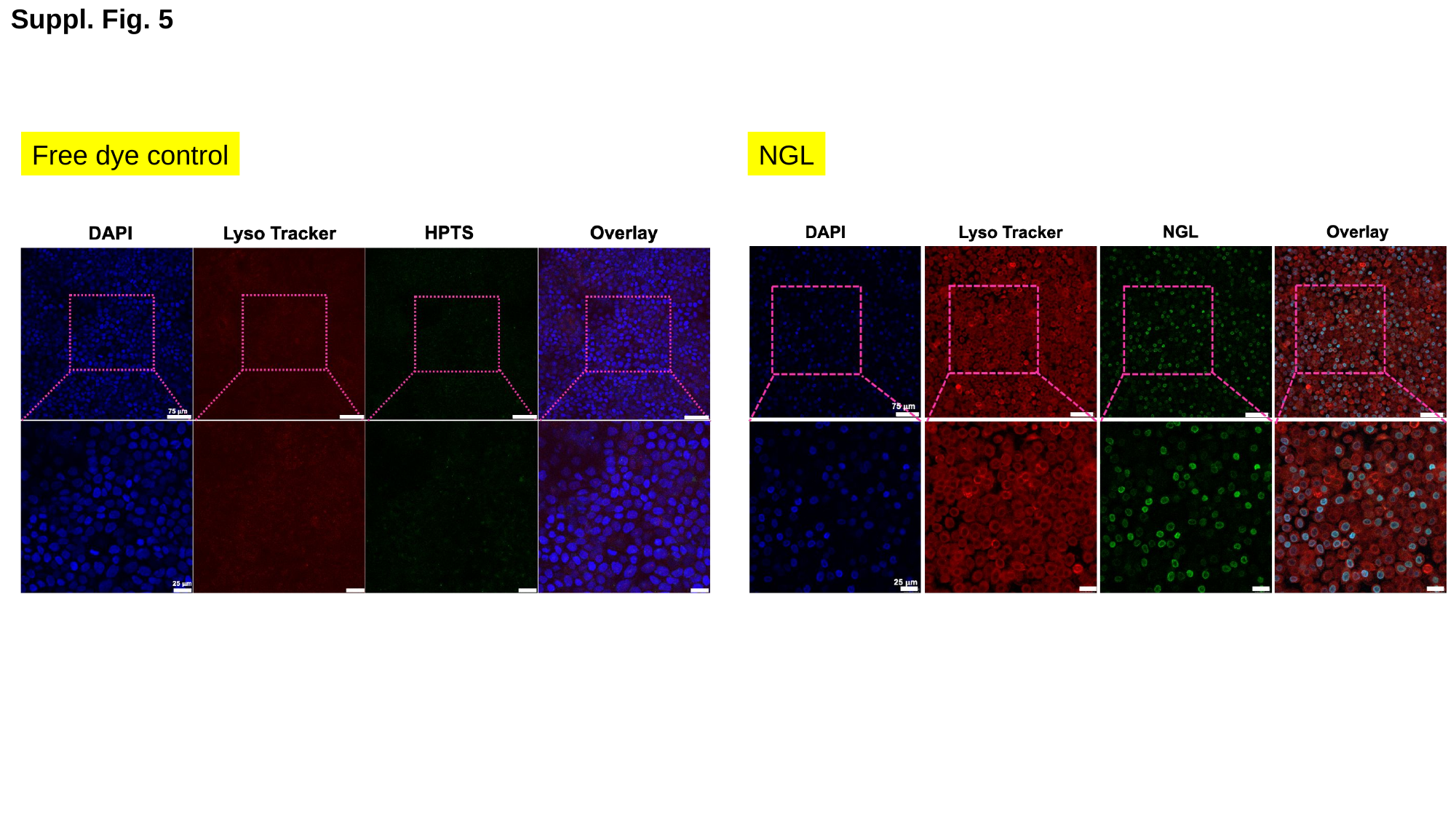

Suppl. Fig. 5
Free dye control
NGL

## Slide 7
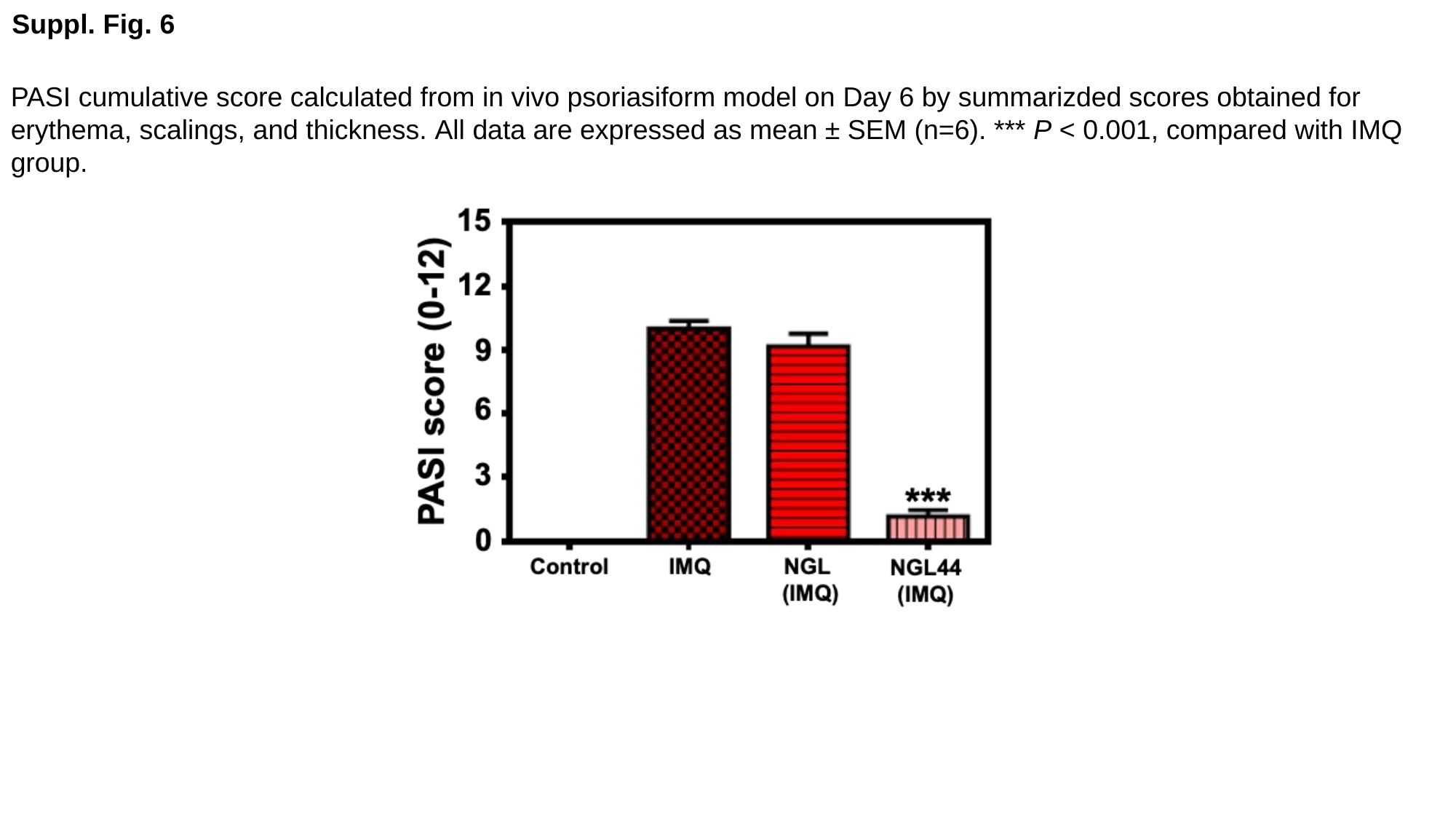

Suppl. Fig. 6
PASI cumulative score calculated from in vivo psoriasiform model on Day 6 by summarizded scores obtained for erythema, scalings, and thickness. All data are expressed as mean ± SEM (n=6). *** P < 0.001, compared with IMQ group.

## Slide 8
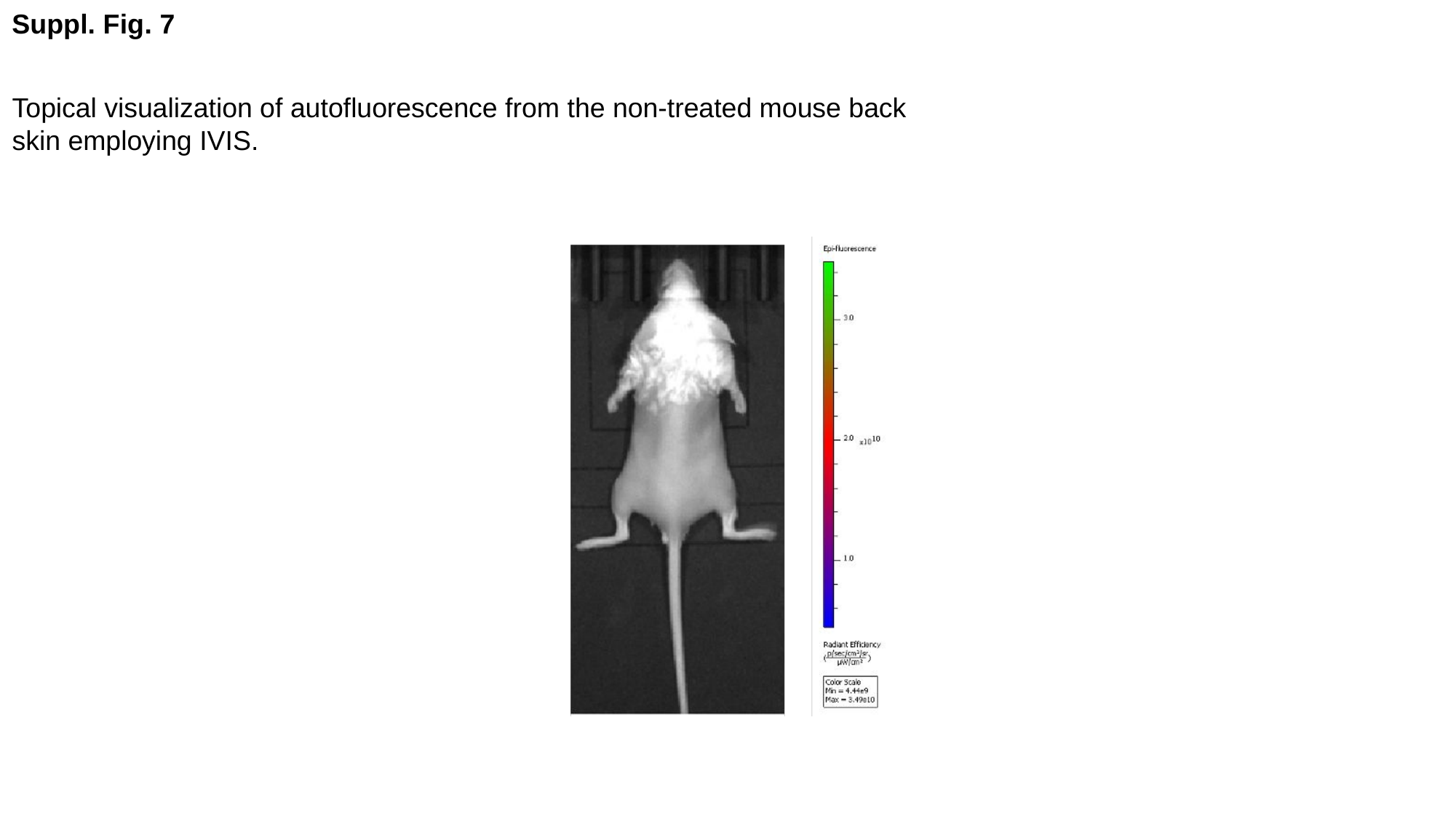

Suppl. Fig. 7
Topical visualization of autofluorescence from the non-treated mouse back skin employing IVIS.
